# Microglia-Derived Extracellular Vesicles from Alzheimer’s Disease Patients Carry miRNAs Driving a Neuroinflammatory Response

**DOI:** 10.1007/s12035-026-05719-w

**Published:** 2026-02-12

**Authors:** Skaiste Arbaciauskaite, Simona Silvestri, Pingyan Luo, Christina Krüger, Zoe Julia Mossmann, Susann Allelein, Alexander Scholz, Dennis Loeffler, Samuele Fiorenza, Anna Menale, Enza Torino, Dirk Kuhlmeier, Oliver Peters, Seija Lehnardt

**Affiliations:** 1 Neuroscience Research Center, Charité-Universitätsmedizin Berlin, Corporate Member of Freie Universität Berlin, Humboldt-Universität Zu Berlin and Berlin Institute of Health, 10117 Berlin, Germany; 2 Fraunhofer Institute for Cell Therapy and Immunology, MicroDiagnostics Unit, 04103 Leipzig, Germany; 3 Interdisciplinary Research Centre On Biomaterials (CRIB), University of Naples Federico II, Naples, Italy; 4 Department of Chemical, Materials and Production Engineering (DICMaPI), University of Naples Federico II, Naples, Italy; 5 Fondazione Istituto Italiano Di Tecnologia, IIT, Naples, Italy; 6 Bioinformatics Unit, Fraunhofer Institute for Cell Therapy and Immunology, 04103 Leipzig, Germany; 7 Next-Generation Diagnostics Unit, Fraunhofer Institute for Cell Therapy and Immunology, 04103 Leipzig, Germany; 8 Department of Information Technology and Electrical Engineering, University of Naples Federico II, Naples, Italy; 9 German Center for Neurodegenerative Diseases (DZNE), Berlin, Germany; 10 Department of Psychiatry, Charité-Universitätsmedizin Berlin, Corporate Member of Freie Universität Berlin, Humboldt-Universität Zu Berlin and Berlin Institute of Health, 10117 Berlin, Germany; 11 Department of Neurology, Charité-Universitätsmedizin Berlin, Corporate Member of Freie Universität Berlin, Humboldt-Universität Zu Berlin and Berlin Institute of Health, 10117 Berlin, Germany

**Keywords:** Microglia, Extracellular vesicles, Alzheimer’s disease, MicroRNA, Toll-like Receptors, EV engineering, RNA delivery

## Abstract

Alzheimer’s disease (AD) represents the most common cause of dementia and urgently requires sensitive biomarkers and effective therapies. Extracellular vesicles represent membranous nano-sized particles secreted from cells, which serve as intercellular messengers participating in central nervous system (CNS) homeostasis, but also are implicated in AD pathogenesis. In addition, EVs containing disease-specific signatures, such as microRNAs (miRNAs), are considered as potent tools for the diagnosis and treatment of AD and other brain disorders. In this study, we used TMEM119 antibody to immunocapture microglia-derived EVs from cerebrospinal fluid (CSF) of AD patients and control subjects. EVs harvested from these CSF samples contained distinct disease-specific miRNA profiles, as assessed by small RNA sequencing. Using a HEK TLR reporter cell system, we found that these miRNA are potent activators of human TLR8, an established RNA sensor. Out of the miRNAs present in AD-associated EVs, selected oligonucleotides were synthesized and loaded into BV2 microglia-derived EVs. Exposure of primary murine microglia to these miRNA-loaded EVs led to TNF release from these cells, thereby driving a neuroinflammatory response. Taken together, putatively microglia-derived EVs from the CSF of AD patients contain miRNAs, which are capable of activating hTLR8 and inducing an inflammatory response from microglia.

**Supplementary Information:**

The online version contains supplementary material available at 10.1007/s12035-026-05719-w.

## Background

Neurodegenerative diseases such as Alzheimer’s disease (AD) pose a substantial global health burden, both in terms of human suffering and economic cost [1]. Despite ongoing research into pathogenic mechanisms, current diagnostic and therapeutic options for these conditions remain limited, highlighting the need for new and more sensitive biomarkers and therapeutic targets.

MicroRNAs (miRNAs), first identified three decades ago as key post-transcriptional regulators [2], have gained prominence for their potential as stable and sensitive biomarkers, with emerging evidence indicating that they also function in immune and cell-to-cell signaling. In particular, miRNAs have been reported as key players in neuroinflammation and neurodegeneration [3–6]. Certain disease-specific miRNAs contain sequences that can directly bind and activate the RNA-sensing immune receptors Toll-like receptor (TLR) 7 and TLR8, thereby inducing an inflammatory response. We have shown in our previous work that *let-7b,* abundantly expressed in the brain, can act as a ligand for TLR7, thereby contributing to neurodegeneration [6]. Moreover, upon binding to TLR7, and also to TLR8, miRNAs can trigger downstream signalling cascades that lead to the production of pro-inflammatory cytokines, such as TNF and IL-6 in the context of neuroinflammatory states [6]. Biofluidic miRNAs are extremely stable in different storage conditions [7]. Therefore, miRNA has been a desirable target for AD’s and other neurodegenerative diseases’ diagnostics and therapeutics.

Extracellular vesicles (EVs) are nano-sized, membranous particles released by various cell types and are found in body fluids [8]. EVs have attracted growing interest for their natural ability to carry different molecular cargo that mirrors the physiological state of donor cells. This cargo can include proteins, nucleic acids, and lipids [9–12]. EVs can cross the blood–brain barrier, showing a dual potential role in neurodegenerative diseases as both diagnostic tools and therapeutic delivery vehicles. Initially described as cell waste transporters, EVs are nowadays recognized to provide an essential cell-to-cell communication mechanism, by delivering molecules that provide useful disease-related information and biomarkers [13]. Due to their protected cargo and stability, EVs can serve as a source of nucleic acid and protein signaling molecules. The EVs’ natural role in intercellular communication, combined with an opportunity to engineer them, positions EVs at the forefront of innovative strategies to enhance immune responses and develop novel diagnostic biomarkers. Microglia-related EVs are discussed as potential key players in neurodegenerative processes since they act as mediators of cell-to-cell communication in both the healthy and the diseased brain [14]. In fact, previous studies have emphasised the influence of EVs on AD progression [15–19]. For example, EVs are involved in Aβ and Tau cell-to-cell spreading and neurotoxicity [19, 20]. On the other hand, microglia-derived EVs can act as neuroprotectors by activating the innate immune response and initiating Aβ clearance in the early stages of AD [21].

Brain-derived EVs can be isolated from CSF [22]. In the case of microglial EVs, so far, no gold microglia-specific surface marker is established. In general, several molecular markers are used to distinguish microglia from other immune and neural cell types. TMEM119, a microglia-specific surface protein, differentiates these cells from macrophages and other cells [23, 24]. CD11b and CD45 expression levels help to identify resting microglia versus macrophages [25–27]. Iba1, a calcium-binding protein, is shared between microglia and macrophages [28, 29]. Similarly, CX3CR [30, 31] and F4/80 [32, 33] are expressed in both microglia and macrophages, with F4/80 present on resting microglia. Activation markers such as CD40 [34, 35], involved in antigen presentation, and CD68, a lysosomal protein, are elevated in activated microglia and macrophages, however, CD68 remains low in resting microglia [36, 37]. The main issue of those markers in EV isolation is that they are not capable of distinguishing resident microglia from circulating blood-derived macrophages. A recent study identified the transmembrane-cell surface protein TMEM119 as the most promising human microglial marker [23].

Here, using TMEM119 antibody we established a protocol to enrich microglia-derived EVs from cerebrospinal fluid (CSF) of AD and control patients. The harvested EV fractions were analyzed for their miRNA cargo by small RNA sequencing. Out of the AD-associated miRNA cargo, selected miRNAs were synthesized and loaded into EVs isolated from murine microglia. Some of these miRNA activated human (h) TLR8 and miRNA-loaded EVs were able to induce cytokine expression in recipient murine microglia. Our findings support a mechanistic role for EV-associated miRNAs in modulating the microglia-driven inflammatory response and may deepen the understanding how endogenous RNA signals may perpetuate neuroinflammation in AD.

## Material and Methods

### Clinical Samples

CSF from 30 patients with AD, frontotemporal dementia (FTD), and healthy controls was obtained from a local multicenter biomaterial bank at Charité – Universitätsmedizin Berlin. Ethical approval for the collection of these samples for the biobank was obtained from the study, which was approved by institutional review boards (Ethikkommission Ethikausschuss 1 am Campus Charité-Mitte, Dementia competence network, [EA1/182/10; BIH CRG 2a, EA2/118/15]). Written informed consent was obtained from all patients participating in the study. Clinical diagnoses were determined in consensus conferences according to DSM-V, considering the patients’ history, neurological and psychiatric findings, neuropsychological test results, CSF biomarkers (t-Tau, amyloid β1–42) and cranial magnet resonance imaging. CSF cut-off values (Aβ1–42 ≤ 600 pg/mL; t-Tau ≥ 350 pg/mL) had previously been determined in-house performing ROC analyses on a data set comprising clinically validated diagnoses (80% for both sensitivity and specificity). To ensure reliability of biomarker values, CSF was collected and analyzed strictly according to protocols. Briefly, lumbar punctures were performed with patients in a sitting position. Exactly 12 mL of CSF were collected in polypropylene tubes. Tubes were shaken, and CSF was centrifuged immediately after collection (1,600 × *g*, room temperature, 10 min), aliquoted (250 μL), and frozen within 30 min after lumbar puncture. The material was stored at –80 °C and was at no time thawed and refrozen. Sampling and storage conditions were identical for samples from all patient groups.

### HMC3 Cells

For the microglial EV surface marker determination experiments with cell culture samples, the human microglial HMC3 (Human Microglia Cells 3, *American Type Culture Collection*) line was used. HMC3 is a human microglial cell line that was isolated from the brain of a patient. This cell line was deposited by KH Krause (University of Geneva, Switzerland). The immortalized human microglial cell line (ATCC® CRL-3304™) is commonly used as an in vitro model to study human microglia biology and neuroinflammation. The transformed human microglial cells retain the properties of primary microglial cells. Cells were cultivated in a humidified atmosphere of 95% at 37 °C and 5% CO_2_ in a *HERA Cell 240i CO2* incubator (Thermo Fisher Scientific, Waltham) and regularly checked for viability and contamination using a *Leica DFC310FX* microscope (Leica Microsystems, Wetzlar). Images of cells were taken using *the Leica Suite software* (Leica Microsystems, Wetzlar) with the following settings: brightness 70% and gamma 0.3. For maintenance, cells were grown in 75 cm^2^ or 175 cm^2^ cell culture flasks (Corning, Corning). HMC3 cells were cultivated in Dulbecco’s modified Eagle’s medium (DMEM) (Gibco, Thermo Fisher Scientific, Waltham) with 10% fetal bovine serum (FCS, Gibco, Thermo Fisher Scientific, Waltham) and 1% penicillin/streptomycin (Thermo Fisher Scientific, Waltham). The medium was refreshed 2–3 times per week, depending on the acidification of the medium, and cells were passaged at a confluence of approximately 80–90% at *a HERA safe laminar flow hood* (Thermo Fisher Scientific, Waltham). In brief, cells were washed with phosphate buffered saline (PBS; Gibco, Thermo Fisher Scientific, Waltham) twice, followed by an incubation of 5–7 min with 0.25% trypsin/EDTA (Gibco, Thermo Fisher Scientific, Bleiswijk) at 37 °C until the cells detached from the surface and separated from each other. To stop the trypsination reaction, complete medium was added, and a homogeneous cell suspension was obtained by pipetting up and down. Cell culture flasks containing pre-warmed complete medium were filled with a portion of the cell suspension for a suitable splitting ratio of 1:3 to 1:8.

### BV2 Cells

The murine microglial cell line BV2 was purchased from Cell Lines Service GmbH (Eppelheim, Germany). Cells were grown in DMEM (High glucose) supplemented with 10% v/v FBS, 1% v/v penicillin/streptomycin and 1% v/v L-glutamine. Sterilized PBS was used for cell washing at a concentration of 10 mM, pH 7.4. Trypsin EDTA Solution (0.5 g porcine trypsin and 0.2 g EDTA) was used for cells detachment. All the media used for cell culture and in vitro studies were purchased from Sigma Aldrich Co. (St. Louis, MO, USA). Cells were incubated at 37 °C in 5% CO_2_/95% air. Cell viability was assessed by trypan blue stain 0.4% (InvitrogenTM, ThermoFisher Scientific; Waltham, MA, USA) and determined using a Countess II FL Automated Cell Counter (ThermoFisher Scientific; Waltham, MA, USA). Dulbecco’s PBS solution used for suspending EVs preparations was purchased from Sigma Aldrich Co. The water used for diluting samples was purified by distillation, deionization, reverse osmosis (Milli-Q Plus, Q-POD®, Merck KGaA, Darmstadt, Germany) and finally filtered with a 0.22 μm cut-off filter.

### Primary Culture of Murine Microglia

C57BL/6 mice were obtained from the FEM, Charité – Universitätsmedizin Berlin, Germany. Animals were maintained and handled in accordance with the German Animal Protection Law and approved by the Regional Office for Health and Social Services in Berlin (Landesamt für Gesundheit und Soziales – LAGeSo, Berlin, Germany). Primary cell cultures of murine microglia were generated as previously described[38]. Briefly, microglia were isolated from mouse brains on postnatal day (P) 1–3. Meninges, superficial blood vessels, hippocampus, and cerebellum were removed from cortices. Cortices were then homogenized with 3 mL Trypsin (2.5%; Gibco #15,090–046, Thermo Fisher Scientific, Waltham, MA, USA) for 25 min at 37 °C. Trypsin reaction was stopped with 4 mL FCS, and 100 μL DNase (Roche #ROD 1284932, Basel, Switzerland) was added for 1 min. Cell suspension was centrifuged at 1200 rpm for 5 min. Pellets were resuspended in DMEM supplemented with 10% FCS and penicillin (100 U/mL)/streptomycin (100 μg/mL), mechanically dissociated, and passed through a 70 μm-cell strainer. Microglia were grown in PDL-coated T75 flasks (Sigma-Aldrich #P0899, Saint Louis, MO, USA) for 10–14 d in 12 mL DMEM at 37 °C in humidified air with 5% (v/v) CO_2_. Microglia were separated from the underlying glial layer by shaking of the flasks for 20 min (300 rpm). Finally, 30,000 microglia/well were seeded in 96-well-plates in DMEM with 10% FCS and penicillin (100 U/mL)/streptomycin (100 μg/mL). On the following day, cells were used for experiments.

### HEK TLR Reporter Cells

HEK Blue™ cells expressing hTLR7 or hTLR8, as well as the respective control cell lines HEK-Blue™ Null1-k and Null1 (InvivoGen, San Diego, CA, USA) were cultured in DMEM (Invitrogen #41,965,062, Carlsbad, CA, USA). DMEM was supplemented with 10% heat-inactivated FCS (Gibco #10,082–147, Thermo Fisher Scientific, Waltham, MA, USA), penicillin (100 U/mL)/streptomycin (100 μg/mL; Gibco #15,140–122, Thermo Fisher Scientific, Waltham, MA, USA) and selection antibiotics Zeocin (100 µg/mL; InvivoGen #ant-zn, San Diego, CA, USA), and/or Blasticidin (10–30 µg/mL; InvivoGen #ant-bl, San Diego, CA, USA).

### Ultrafiltration

Before starting HMC3 cell EVs’ ultrafiltration, ultrafiltration filters (Centricon-Plus 70, Ultracell Pl – 10, 10.000 NMWL (Merck Millipore, Burlington)) were rinsed twice with 30 PBS (Gibco, Thermofisher) with 1% (v/v) *Tween® 20* (PBST, Carl Roth, Karlsruhe). Next, the supernatant of HMC3 cells was concentrated by centrifugation at 3,500 *RCF,* 4 °C. The centrifugation time depended on the original volume and the concentration factor (longer centrifugation time resulted in higher concentration). After centrifugation, an up-side-down spin of 2 min at 1,000 RCF*,* 4 °C was done to collect the concentrated supernatant. The supernatant was stored in aliquots at −80 °C.

### Immunomagnetic Bead-Based Isolation of EVs

Immunomagnetic bead‑based isolation of EVs was performed with *Dynabeads™ MyOne™ Epoxy* (Thermo Fisher Scientific, Waltham, MA). Lyophilised beads (60 mg total; 2 mg per reaction) were equilibrated to room temperature, hydrated in 1 mL Buffer A, vortexed for 30 s and washed twice on a magnetic rack (1–2 min each). Washed beads (2 mg in 240 µL Buffer A) were conjugated with either TMEM119 antibody (18 µL per 2 mg beads; A16075D, BioLegend) to enrich microglia‑derived EVs, or CD63 antibody (NVG2, BioLegend) as an EV positive control. PBS was added to bring the antibody volume to 40 µL, and the suspension was brought to a 1:1 ratio with Buffer B (final volume 480 µL). Conjugation proceeded overnight (16–24 h) at RT with gentle tilt rotation. Antibody‑coupled beads were then recovered magnetically, washed four times with 1 mL PBS (2 min each), and blocked for 1 h at RT in 0.5 mL PolyAn blocking solution. For EV capture, 2 mg of blocked beads were added to 0.5 mL cerebrospinal fluid (CSF) diluted 1:3 with PBS (final volume 2 mL) and incubated for 1 h at RT with slow rotation. Beads were collected on the magnet for 4 min, washed three times with 1 mL PBST (0.05% Tween‑20, 2 min each), and finally resuspended in 100 µL PBS for downstream analysis. Dynabeads were magnetically retained in a rack during all separation and washing steps.

### SDS-PAGE and Western Blot

For protein separation, hand-casted or commercially available stain-free – 20% (BioRad Laboratories, Hercules) SDS-PAGE gels were used. In-house gels were prepared as follows: separation gel of 12% acrylamide (Carl Roth, Karlsruhe) was cast and polymerized before the stacking gel of 5% acrylamide was layered on top and the comb for subsequent sample loading slots was inserted. After polymerization, the comb was removed, and the slots were rinsed with Towbin buffer (25 mM TRIS base; 192 mM glycine; 0.1% (w/v) SDS). Samples were prepared with 6 × Laemmli buffer, heated for 10 min at 90 °C prior to electrophoresis. Protein separation according to size was performed in 2 steps starting from 70 V for 30 min, followed by 120 V for 100 min in Towbin buffer in a *Mini-Protean® Tetra Vertical Electrophoresis Cell* (BioRad Laboratories, Hercules). After electrophoresis, total protein staining was observed directly in the stain-free gel after fluorescent activation using the *ChemiDoc MP* device (Bio-Rad Laboratories, Hercules). Subsequently, the gel was washed twice with ddH2O and equilibrated in cathode buffer for membrane transfer. On a *Trans-Blot® SD semi-dry transfer cell* (BioRad Laboratories, Hercules), a sandwich of extra thick filter paper and a nitrocellulose membrane (0.2 μm, GE, Boston) both soaked in anode buffer were placed, followed by the equilibrated gel and another extra thick filter paper soaked in cathode buffer. Transfer of the proteins from the gel to the membrane was achieved at 110 mA for 60 min. The membrane was blocked in 5% (w/v) BSA in PBST for 1 h at RT. Incubation with the primary CD81 antibody (anti-human CD81 (TAPA-1), 5A6, Biolegend), 1:1,000, was conducted overnight at 4 °C under constant agitation. Incubation with the primary antibody was finished with 4 washing steps of 5 min in PBST at RT. After the washing steps, the incubation with the secondary HRP-conjugated antibody (goat IgG anti-Mouse IgG-HRPO, Dianova) 1:10,000 for 1 h at RT was performed. Two washing steps, each for 5 min, with PBST and then with PBS were conducted, before the *Clarity (Max) Western ECL substrate* (Bio-Rad Laboratories, Hercules) was applied. Signals were analyzed in *the ChemiDoc MP* device with a suitable exposure time depending on the signal intensity. When analyzing more than one marker after immunomagnetic isolation, the membrane was washed with PBST for 5 min at RT, followed by an incubation with the primary antibody and the subsequent procedure as described above.

### RNA Isolation from EVs

After immunomagnetic isolation from human CSF, the magnetic beads dissolved in 200 μL PBS were lysed by applying 800 μL *TRIzol™ LS* (Thermo Fisher Scientific, Waltham). 200 μL chloroform were added, and the mixture was shaken vigorously by hand for 15 s and left for 3 min at RT. Subsequent centrifugation for 15 min at 12,000 RCF (Eppendorf Centrifuge 5424 R) formed three layers of which only the top aqueous layer of 400 μL was transferred into a new tube. Subsequently, 1350 µL of 100% ethanol was added, left for 5 min at RT, and then was centrifuged for 20 min at 14,000 RPM, 4 °C. After centrifugation, 100% ethanol was removed, and 1 mL 70% EtOH were added. Subsequent centrifugation for 5 min at 9,500RPM, 4 °C was conducted. The supernatant was removed, and complete drying of the pellet was permitted for 30 min at 40 °C prior to resuspension in nuclease-free water.

### Small RNA Sequencing

EV RNA isolated from 27 samples was analyzed by small RNA sequencing. To this end, EV RNA was resuspended in 12 μL RNase‐free water. For subsequent RNA sequencing analysis, 8 µL RNA per sample were used. Library preparation was performed using D‐Plex Small RNA‐seq Kit v2 (Diagenode C05030001) and D‐Plex Single Indexes for Illumina–Set A (Diagenode C05030010) according to the manufacturer's protocol. After size selection of the libraries by a 4% agarose gel (~ 195 bp expected), they were quantified using a Qubit DNA kit and the DeNovix instrument (Biozym). Finally, the size distribution was determined on a Bioanalyzer 2100 instrument using a high sensitivity DNA assay (Agilent Technologies). The molarity of each library was calculated and equal amounts were pooled. Finally, sequencing of 151 bp single read was performed using the NextSeq 2000 P2 Reagent Kit (200 cycles) on a NextSeq 2000 instrument (Illumina). The final molarity of the library pool on the sequencing flow cell was 650 pM, including 1% PhiX library.

### Bioinformatic Data Analysis

Reads were demultiplexed by Illumina’s bcl-convert (v3.9.3). For a reproducible data analysis the workflow manager uap (v2.2.0)[39] was used. Since the D-Plex Small RNA-seq Kit (V2 03_2021) of Diagenode was used an analysis pipeline based on the recommended protocol was applied. At first fumi_tools (v0.18.2) with subcommand copy_umi and parameter –threads 10,–umi-length 12 was used to process the UMIs. The adapter sequences were removed from reads by Cutadapt (v3.5)[40] using the recommended parameters –trim-n, –nextseq-trim 20, –error-rate 0.2, –times 4, –match-read-wildcards, –minimum-length 15, –cut 16, –a AGATCGGAAGAGCACACGTCTG, -a AAAAAAAA, -a GAACTCCAGTCAC). Bowtie2 (v2.4.4) with parameters –norc, –threads 6, -N 0, -L 15 was used to align reads against the mirbase (release 22.1). SAMtool (v1.14)[41] converts the alignment to a by-name sorted BAM file. fumi_tools (v0.18.2) with subcommand dedup was used for the UMI deduplication. Finally, number of reads per gene were counted by featureCounts from Subread (v2.0.3) [42] using parameters -t miRNA, -g Name, -s 1, -R CORE. The further analysis was performed using R (v4.2.2). Venn diagramms were created by using the package VennDiagram (1.7.3) [43]. GO data was retrieved using biomaRt (2.54.1) [44].

### Microfluidization for High-Throughput EVs Production

BV2-derived EVs were obtained using an established method, which employs a dynamic high-pressure stimulation to microfluidize lipidic entities [45, 46]. In detail, BV2 cells were initially seeded in a 150 cm^2^ culture flask. Once they reached the desired confluency, 7 × 10^6^ cells were collected and suspended in cold, 0.22 µm-filtered PBS. This cell suspension was processed in a bench-top High-Pressure Homogenizer (HPH) equipped with an ice-cooled interaction valve and a thermocouple to ensure that the temperature was kept below 10 °C throughout the process and subjected to 10 cycles at 1,000 bar. During microfluidic processing, cells were forced through a Y-shaped microfluidic valve, where high shear, elongational, cavitation, and collision forces combined to create a complex force field that promoted a high-throughput vesicle secretion. Subsequently, purification by differential ultracentrifugation was performed to isolate the vesicles from cells and other contaminants. To this end, the processed suspension was first centrifuged at 300 × g for 10 min at 4 °C (F15-6 × 100y ROTOR) using an SL 16R centrifuge to remove dead cells, followed by centrifugation at 2,000 × g for 10 min at 4 °C and 10,000 × g for 30 min at 4 °C to remove cellular debris. The vesicle-containing suspension was then transferred to polycarbonate tubes (6.5 mL, 16 × 64 mm, Thickwall, Beckman Coulter, Brea, CA, USA) and ultracentrifuged twice at 110,000 × g for 70 min at 4 °C using an Optima™ MAX-XP Ultracentrifuge (Beckman Coulter, Brea, CA, USA). After each ultracentrifugation step, the supernatant was discarded, and the vesicle pellets were resuspended in 0.22 µm-filtered PBS. Finally, purified vesicles were resuspended in a final volume of 200 µL and stored at 4 °C for short-term storage or at −20 °C and −80 °C for mid- and long-term storages, respectively.

### Morphological and Physico-Chemical Characterization of HPH Cell-Derived EVs

The morphological characterization of EVs secreted by HPH-stimulated cells was performed by combining the Nanoparticle Tracking Analysis (NTA) size distribution measurements and transmission electron microscopy (TEM) analysis. For the determination of the sample concentration and the analysis of the size distributions, samples were tested by the NTA using Zetasizer Nano ZS (Malvern Panalytical, UK) in biological triplicates by diluting 1:1,000 each vesicles’ preparation thus ensuring that 20—100 particles were visible in the field of view. Three runs of 60 s per sample were recorded at 25 °C with a manual shutter adjustment and the screen gain and camera level set at 1 and 16, respectively. After that, pre-processing data was performed by setting a detection threshold at 3–5, and results were analyzed with the NTA Tracking Analysis software. The morphological analysis was performed by dropping 5 μL of the purified sample on a Formvar/Carbon 200 mesh grid (Agar scientific) and observed at an 80 kV accelerating voltage with a TEM TECNAI (by FEI) instrumentation. Zeta potential measurements were also performed at a temperature of 25 °C on the Zetasizer Nano ZS loading the high-concentration surface zeta potential cell (Malvern Panalytical, UK) with 1 mL of the NP suspension.

### Immunofluorescence Analysis by Leprechaun System

To characterize and quantify the biological content, the obtained EVs population was analyzed through the Leprechaun® system, whose working principle is based on immunocapture and fluorescence detection, according to the manufacturer’s instructions (Unchained Labs). In detail, Luni chips were first pre-coated with the primary antibodies CD9, CD63, and CD81 (Leprechaun Exosome Human Tetraspanin Kit), which are common surface markers for EVs. Then, a first background measurement was performed, while samples were diluted in 1X incubation solution at 1:2 so that concentrations of EVs could fall in the range [10^5^–10^9^] particles/mL, corresponding to the sensitivity limit of the instrument, and 50 μL of diluted samples were incubated for 16 h at RT. Subsequently, chips were washed twice, incubated with fluorescence-labeled secondary antibodies for 1 h (1:500 each of anti-CD9 CF488A, anti-CD81 CF555, and anti-CD63 CF647), and dried using the Chip Washer (Unchained Labs). Immediately after drying, chips were loaded onto the Leprechaun instrument and analysed using Leprechaun® Analyser 1.0 software (Unchained Labs) specific for the interferometric imaging and fluorescence analysis.

### EV Engineering by Microfluidics

BV2-derived vesicles were loaded with the selected synthetic miRNA candidates through a hyperbolic extensional microfluidic device designed and fabricated by micromilling technique. First, the 2.5 × 0.12 × 10 mm device geometry was designed through AutoCAD software (didactic version T.72.0.0 AutoCAD 2023.0.1) and then converted into the computer-aided manufacturing (CAM) files for the fabrication by using DESKAM 2000 (version 5.1.5.11) software. A PMMA slab (1.1 mm thickness, product code ME30-SH-000110, GoodFellow) was used as material for microfluidic device fabrication. The polymer was processed through a Minitech CNC mini-mill/GX (Minitech Machinery Corp., USA) machine to obtain the designed geometry. An endmill tip having a diameter of 100 μm was used to engrave the channels, with an X–Y step of 0.05 mm, a Z step of 0.024 mm, a spindle rate of 10,000 rpm, and a feed rate of 10%. The final microfluidic device was obtained by bonding the bottom micro-machined PMMA slab (containing the channel) and the superior lid, by casting IPA 70% (v/v) in between, the two PMMA slabs were compressed by means of clamps and then cured at 68 °C for 15 min. A total volume of 1.2 mL obtained by mixing 400 µL of vesicle suspension with 75 µL of miRNA and 725 µL of PBS was loaded in a 5 mL plastic syringe (BD PlastiPak™ Syringe with Luer Slip), mounted on a Harvard Syringe Pump and connected to the microfabricated device, following the stimulation at a FR = 1 mL/min. After processing, a final volume of 1 mL was collected, quantified at Nanodrop, and exploited for downstream assays.

### MiRNA Oligoribonucleotides and TLR Agonists

MiRNA oligoribonucleotides were synthesised by Integrated DNA Technologies. All oligoribonucleotides contained phosphorothioate bonds, depicted below as “s”. The sequences of the synthetic miRNAs were, as follows:

- *let-7e*−5p, 5‘-UsGsAsGsGsUsAsGsGsAsGsGsUsUsGsUsAsUsAsGsUsU-3‘;
- miR-3150b-3p, 5′-UsGsAsGsGsAsGsAsUsCsGsUsCsGsAsGsGsUsUsGsG-3;
- miR-548b-3p, 5′-CsAsAsGsAsAsCsCsUsCsAsGsUsUsGsCsUsUsUsUsGsU-3′;
- miR-154-5p, 5′-UsAsGsGsUsUsAsUsCsCsGsUsGsUsUsGsCsCsUsUsCsG-3′.

For all experiments with HEK TLR reporter cells and microglia, miRNA mimics were complexed and transfected with Lyovec (Invivogen, #lyec-1). Loxoribine, LPS, R848, and TNF were purchased from Invivogen (San Diego, CA, USA).

### Enzyme-Linked Immunoabsorbent Assay

Primary mouse microglia cells were incubated with 50 µL of EVs loaded with miRNA, as indicated in the respective figure, together with 100 µL of culture medium for 24 h. Subsequently, supernatants were collected and stored at −80 °C. TNF and IL-6 concentrations in the supernatants were measured by Enzyme-Linked Immunoabsorbent Assay (ELISA) according to the manufacturer’s instruction (TNF alpha Mouse Uncoated ELISA Kit, Invitrogen, #88–7324–88, Carlsbad, CA).

### Statistics

All data are presented as mean ± SEM. For Figs. 5A and 5B, statistical differences across experimental groups were assessed using a two-tailed Dunnett’s test to compare each treatment condition against a single control group. Dunnett’s test controls the family-wise error rate for multiple comparisons by adjusting p-values based on the joint distribution of test statistics. For Fig. 8, comparisons between two experimental groups were performed using unpaired, two-tailed Student’s t-tests assuming unequal variances. To account for multiple testing across several variable pairs, raw p-values were adjusted using the Benjamini–Hochberg procedure to control the false discovery rate (FDR). For all figures, significance thresholds were defined as follows: *p < 0.05; **p < 0.01, ***p < 0.001. ns (not significant). Statistical analysis and data visualization were performed using Python 3.9.7 with SciPy 1.13.1, Matplotlib 3.6.0, and Statsmodels 0.14.4.

## Results

### TMEM119-Based Isolation of Microglial EVs from CSF of AD Patients and Control Individuals

**Fig. 1:**
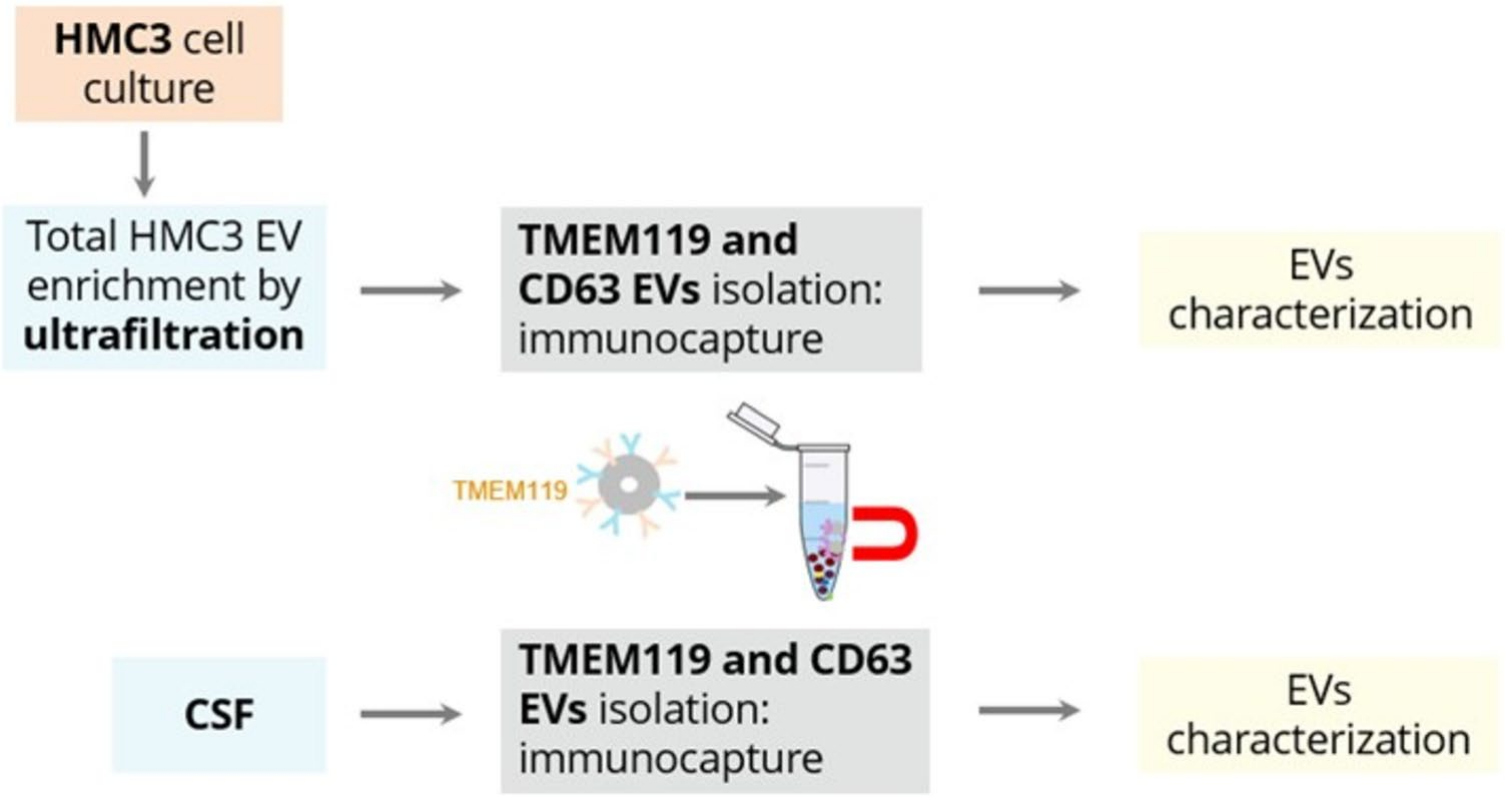
Workflow of experimental steps of TMEM119-based immunocapture of microglial EVs from HMC3 cells and human CSF**.** For isolation of EVs derived from HMC3 cell culture, the total EV fraction was first enriched by ultrafiltration. Immunocapture was performed using magnetic beads conjugated with antibodies targeting TMEM119 as microglia-specific marker and CD63, a well-established general EV marker

TMEM119 is expressed in immortalized human microglia, in which the expression levels were not affected by exposure to lipopolysaccharide (LPS), IFN-γ, IL‐4, IL‐13, or TGFβ1. Notably, TMEM119 immunoreactivity was previously detected exclusively on a subset of Iba1^+^ CD68^+^ microglia with ramified and amoeboid morphology in the brains of neurodegenerative diseases, including AD, whereas Iba1^+^ CD68^+^-infiltrating macrophages do not express TMEM119 in demyelinating lesions of multiple sclerosis and necrotic lesions of cerebral infarction [23]. We sought to test TMEM119 antibody as a microglia-specific surface marker to identify and isolate microglia-derived EVs. To this end, we employed a systematic workflow beginning with culturing the HMC3 cell line. This initial step ensured that the later harvested EVs sourced exclusively from microglia, thereby reducing potential contamination with any other (CNS) cell types. Following cultivation of HMC3 cells, we isolated and concentrated their EVs by ultrafiltration (Supplemented materials 1). Subsequently, immunomagnetic bead-based isolation was used to capture EVs from HMC3 ultrafiltrates. After successful immunocapture from HMC3 cells, in a second step, TMEM119 antibody was used to capture EVs from human cerebrospinal fluid (CSF) samples. In detail, epoxy beads were conjugated with either TMEM119 antibody to enrich microglia-derived EVs, or CD63 antibody as a general EV marker. Following antibody coupling and blocking, as described in the *Methods* section, the beads were incubated with diluted CSF samples derived from patients diagnosed with AD, frontotemporal dementia (FTD), or from control individuals. FTD samples were included as an additional neurodegenerative disease comparator to distinguish AD-associated patterns from alterations potentially shared across neurodegeneration. A scheme of the experimental steps, leading to microglia-derived EV isolation from HMC3 cells and human CSF, is shown in Fig. 1.

**Fig. 2:**
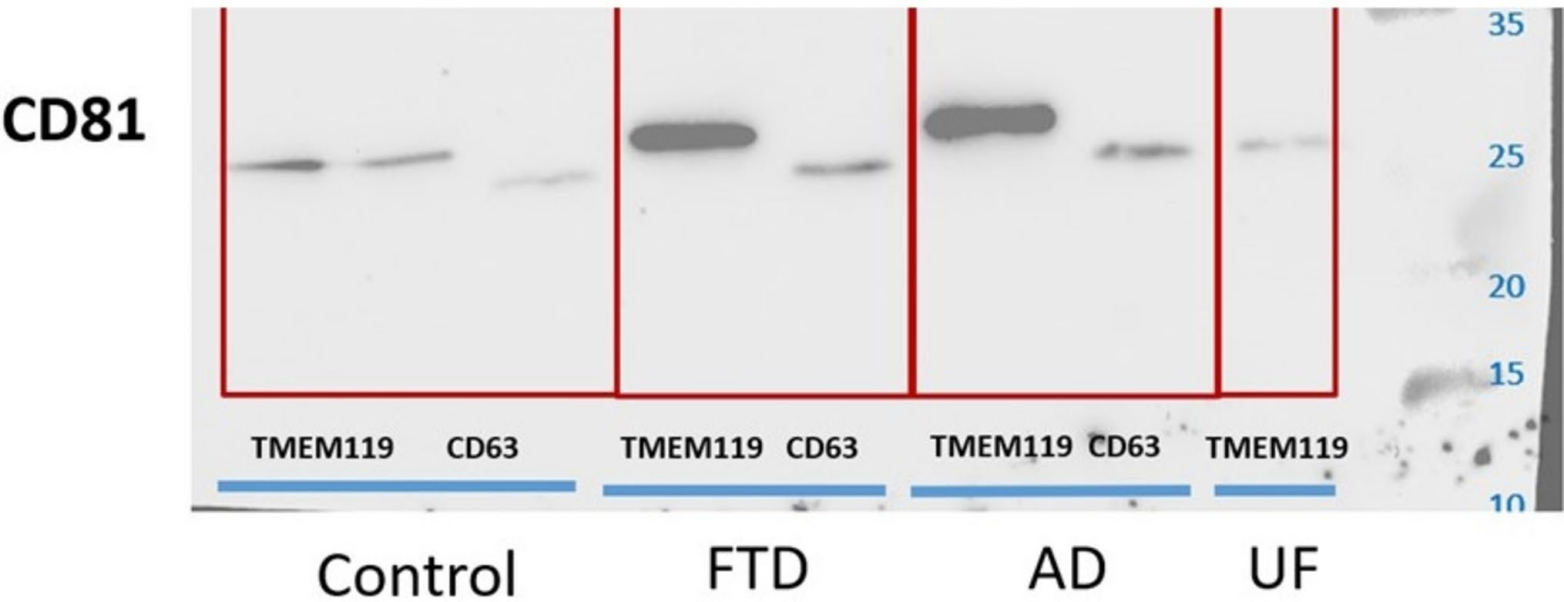
Validation of EV enrichment using immunomagnetic isolation targeting TMEM119 and CD63**.** To confirm the presence of EVs following immunomagnetic capture using anti-TMEM119 and anti-CD63 antibodies, western blot analysis employing an antibody directed against CD81, a general EV marker, was performed. EVs isolated from CSF samples of patients with FTD, AD, and healthy control subjects (control) were tested, as indicated. EVs isolated from HMC3 cell culture supernatant enriched by ultrafiltration (UF) served as positive control for EV presence. CD81 expression was detected in all samples, confirming successful EV enrichment across conditions

After the immunomagnetic isolation step, EV fractions captured from AD CSF, FTD CSF, and control CSF using TMEM119-conjugated beads, as well as EV fractions captured using CD63-conjugated beads as a general EV-associated reference, were analysed by western blot. HMC3 lysate served as a positive control. The ubiquitous EV-associated marker CD81 was detectable in the immunocaptured fractions, confirming the presence of EV-associated material in the isolates (Fig. 2). Because EV input and loading were not normalized across lanes (e.g., by particle number or total EV protein), therefore the band intensities were not quantified, and the CD81 blot is presented as qualitative/confirmatory evidence rather than a quantitative comparison between diagnostic groups. We therefore refer to these isolates as TMEM119-enriched EV fractions (putatively microglia-associated) obtained from human CSF.

### Identification of miRNA Cargo of Microglia-Derived EVs from AD and Control CSF

**Fig. 3:**
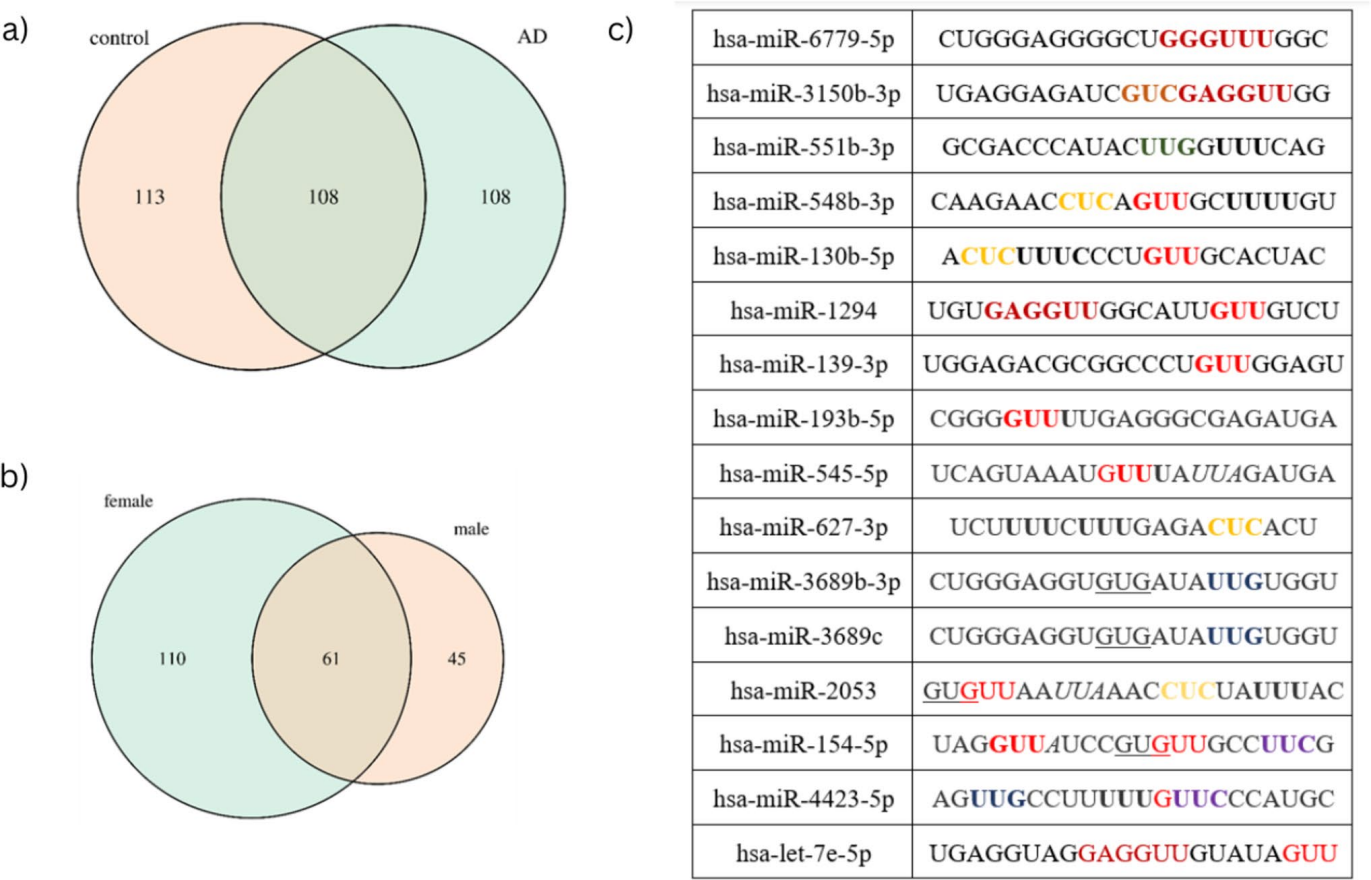
Differential analysis and functional prediction of miRNAs associated with microglia-derived extracellular EVs in AD**.** This figure summarizes the identification, TLR-binding prediction, and functional characterization of EV-associated miRNAs isolated from CSF of AD patients and healthy controls. EVs were enriched by immunocapture with anti-TMEM119 and anti-CD63 antibodies to selectively isolate microglia-derived vesicles. Small RNA sequencing was then performed to profile miRNA content. (**a**) Venn diagrams show the number of distinct miRNAs identified in EVs from AD patients compared to controls (top), and (**b**) sex-based differences in miRNA profiles within the AD group (bottom). (**c**) Out of the miRNAs found in AD EVs miRNA candidates were selected and analyzed regarding their potential to activate TLR7 and TLR8 using the *BrainDead* algorithm

Having validated TMEM119 as a robust antibody tool to isolate putatively microglia‐derived EVs from human CSF, we next applied this EV isolation strategy to analyze and compare the miRNA cargo of CSF samples from AD patients and control subjects. In detail, 15 CSF samples from AD patients and 12 CSF samples from age-matched control subjects were analyzed. At this stage, we did not include FTD samples due to the limited availability of well-characterized FTD CSF specimens. After microglia-derived (TMEM119^+^) EVs were successfully isolated from the 27 CSF samples named above, small RNAs from each EV fraction were enriched. Small RNA sequencing revealed distinct miRNA cargo profiles in microglia-derived EV fractions derived from AD patients versus control subjects. A total of 221 features were detected in the control EV fractions (among them, 113 were unique to control condition) and a total of 216 features were identified in the AD EV fractions (among them, 108 were unique to AD condition), with 108 features common to EV fractions isolated from both groups (Fig. 3a). Furthermore, the total miRNA profiles were analyzed regarding sex differences. Within the AD group we found a differential pattern between female and male samples: a total of 171 features were detected in females (110 unique to females) and 106 in males (45 unique to males), with 61 features shared between both sexes (Fig. 3b).

Next, we tested for differential expression between the AD and control EV fraction groups. The miRNAs found differentially and specifically expressed in the AD EV fraction were analyzed by the machine learning approach *BrainDead*, which predicts short single-stranded RNAs as potential activators of TLR7/8 based on sequential and structural features [47]. The top ranked miRNA candidates (hsa-let-7e-5p (rank 24/2656), hsa-miR-154-5p (rank 51/2656), hsa-miR-3150b-3p (rank 201/2656), hsa-miR-548b-3p (rank 261/2656) predicted to serve as potent TLR7 and TLR8 ligands are given in Fig. 3c.

### Microglial EV-Associated miRNAs Activate Human TLR8

**Fig. 4:**
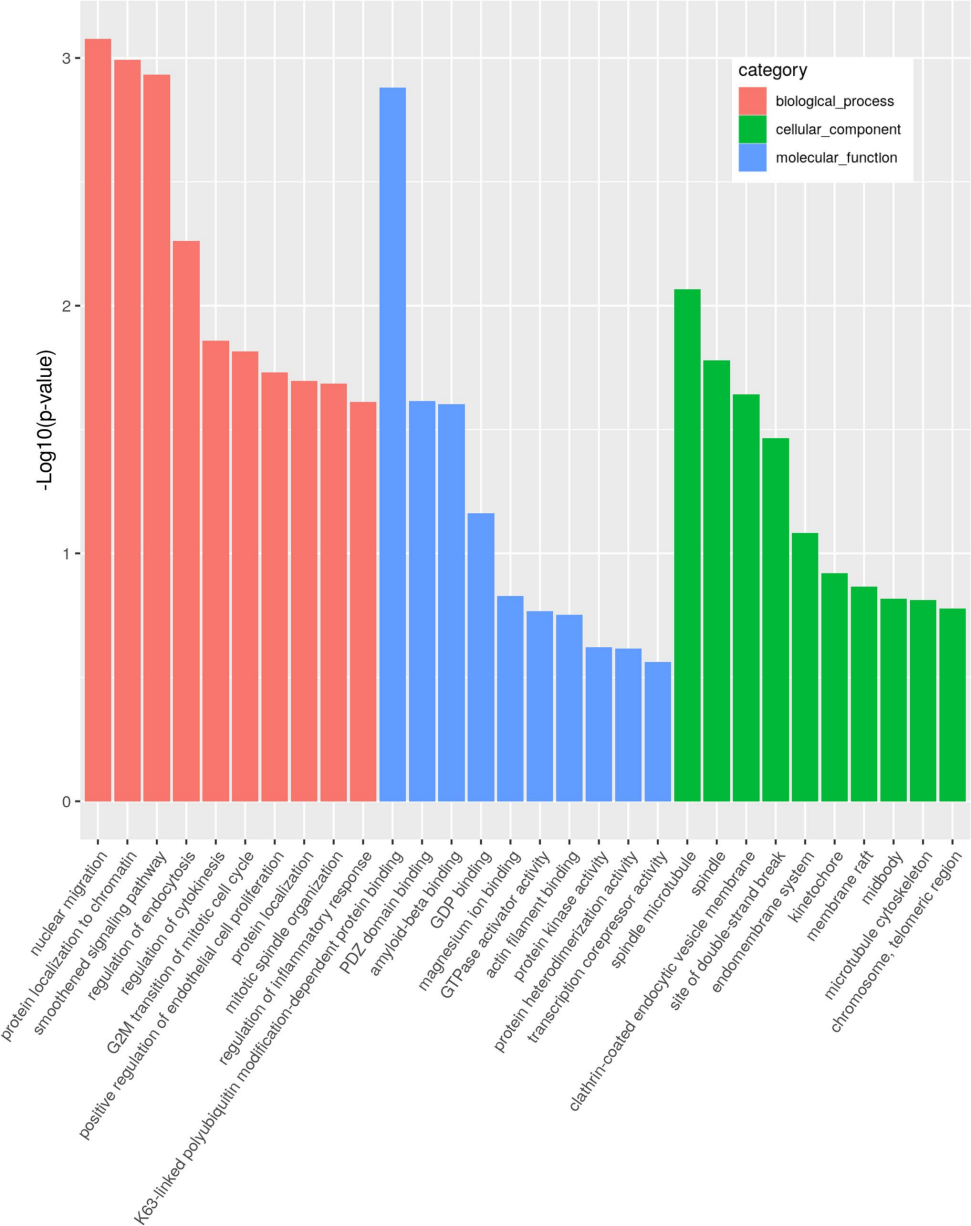
Gene Ontology (GO) enrichment analysis. GO enrichment analysis was conducted on the final set of candidate miRNAs (miR-3150b-3p, miR-548b-3p, miR-154-5p and *let-7e*) to identify biological processes potentially regulated by their target genes. Over-represented GO terms were grouped into three categories based on their relevance to AD pathology: (1) biological process (red), (2) cellular component (green), and (3) molecular function (blue)

Out of the miRNAs predicted as potential TLR7/8 ligands by *Braindead*, the candidate miRNAs, namely miR-3150b-3p, miR-548b-3p, miR-154-5p, and *let-7e* were shortlisted and further investigated by functional assays based on their nucleotide composition, which resembled GU-rich motifs known to be recognized by TLR7/8 [47]. In parallel, Gene Ontology (GO) enrichment analysis of predicted miRNA targets provided complementary context. We performed analysis of putative target genes regulated by miR-3150b-3p, miR-548b-3p, miR-154-5p, and *let-7e*, narrowing the analysis down to three functional categories: (1) neurodegeneration-related pathways, (2) inflammation and microglial activation, and (3) synaptic function, mitochondrial dynamics, and autophagy (Fig. 4). Collectively, our enrichment analysis suggests that the miRNA candidates we tested (miR-3150b-3p, miR-548b-3p, miR-154-5p, and *let-7e*) operate at the interface of neurodegeneration, neuroinflammation and cellular homeostasis. As shown in Fig. 4, their predicted targets are significantly enriched for GO terms related to amyloid‑β binding, regulation of inflammatory responses, cytoskeletal and vesicle‑trafficking processes (e.g. endocytosis, spindle and microtubule organization), and chromatin/cell‑cycle control. These functions are highly relevant to pathways implicated in neurodegenerative disease pathophysiology.

**Fig. 5:**
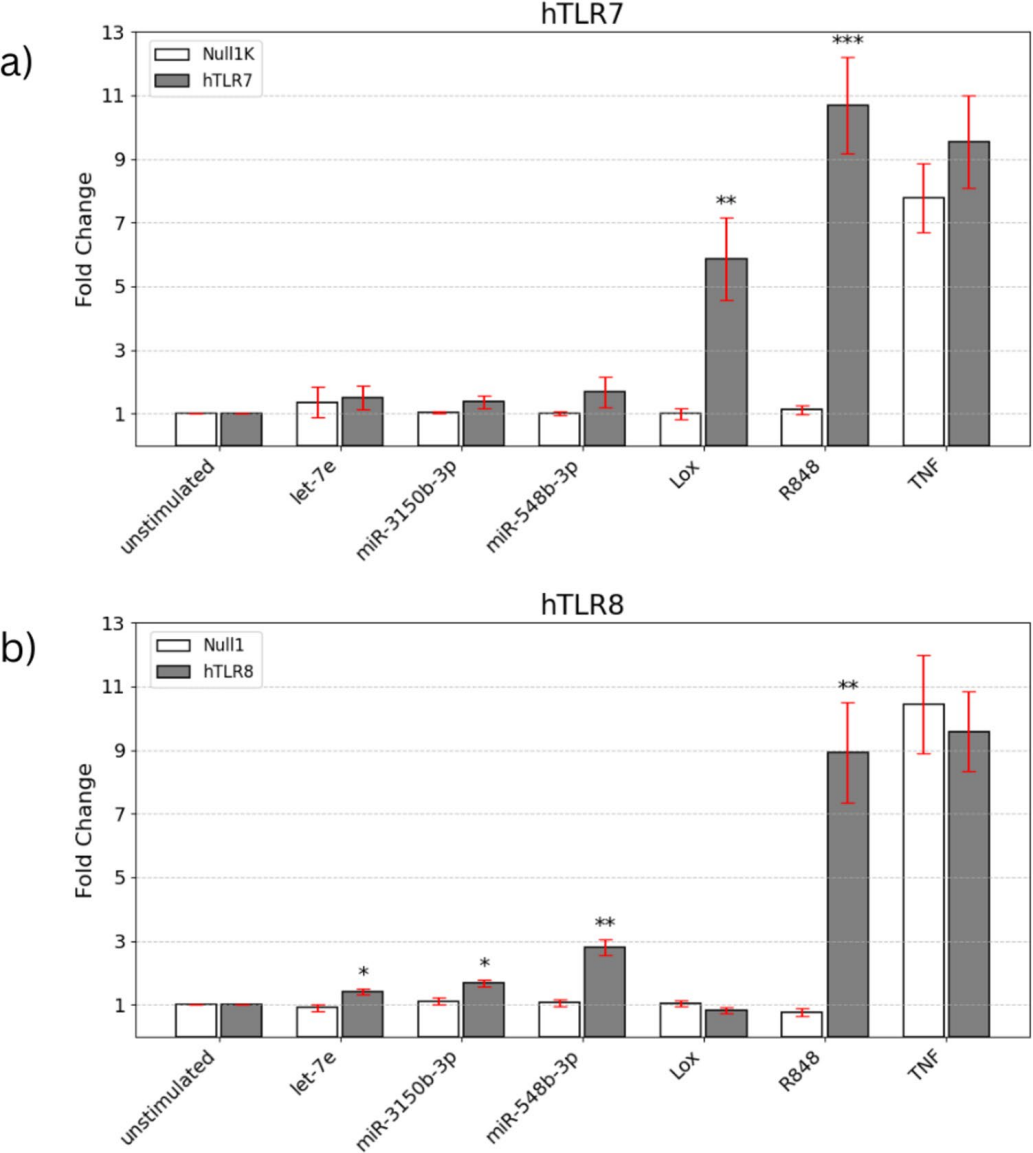
AD EV-associated miRNAs activate hTLR8**.** HEK293-derived reporter cells expressing hTLR7 (**a**) or hTLR8 (**b**), and their corresponding parental control lines Null1K and Null1, were stimulated with 10 μg/mL of miRNA mimics *let-7e*, miR-3150b-3p, and miR-548b-3p, as indicated, for 24 h. Loxoribine (1 mM) and R848 (10 μg/mL) served as positive control for TLR7 and TLR7/8 activation, respectively. TNF (0,1 μg/mL) served as positive control for HEK293-derived cell line activation. Unstimulated cells served as negative control. Results are expressed as mean ± SEM. *n* = 3. Significance was determined using Welch’s two-sided *t*-test adjusted for multiple comparisons. The vertical axis represents fold change. *p < 0.05; ** *p* < 0.01; *** *p* < 0.001

To validate the role of miR-3150b-3p, miR-548b-3p and *let-7e* as ligands for hTLR7 and hTLR8, we employed a synthetic transfection agent to introduce the miRNA of interest into HEK-Blue™ hTLR7 or hTLR8 reporter cells. The parental cell lines HEK-Blue™ Null1-k and Null1 served as the respective negative control cell lines. miR-154-5p was not included in this TLR reporter cell assay because its potential to activate hTLR7, hTLR8, and mTLR7 has already been reported in our previous study [48]. After 24 h miRNA incubation, the ability of *let-7e*, miR-3150b-3p, and miR-548b-3p to activate hTLR7 (Fig. 5a) and hTLR8 (Fig. 5b) were assessed by HEK-Blue Secreted Embryonic Alkaline Phosphatase (SEAP) activation. None of the tested miRNA candidates significantly activated hTLR7. In contrast, both the established TLR7 agonist loxoribine and the dual TLR7/8 agonist R848 elicited receptor activation in hTLR7-expressing reporter cells, as expected. In TLR8-expressing reporter cells, however, a different pattern emerged (Fig. 5b). All three miRNA candidates, namely miR-3150b-3p, miR-548b-3p, and to a lesser extent *let-7e*, induced a receptor response. Also, R848 induced a receptor response, as expected. The parental control line did not respond to any of the tested miRNAs, nor to R848, confirming receptor-specificity of the observed miRNA-induced effects on hTLR8-expressing reporter cells (Fig. 5b). In both, hTLR7- and hTLR8-expressing reporter cells TNF served as the established positive control for cell activation. It is expected to increase reporter activity in all of the HEK-Blue™ SEAP reporter cell lines by signaling through TNF receptors, and activating NF-κB/AP-1. This effect is independent of TLR expression, so both the TLR7/8-overexpressing and the Null parental control lines are expected to respond, and this effect was observed. Overall, these data demonstrate that extracellularly applied miR-3150b-3p, miR-548b-3p and *let-7e* are capable of activating hTLR8, while failing to elicit a comparable response through hTLR7.

### Boosting Biogenesis of BV2-Derived EVs and miRNA Encapsulation by Microfluidization

**Fig. 6:**
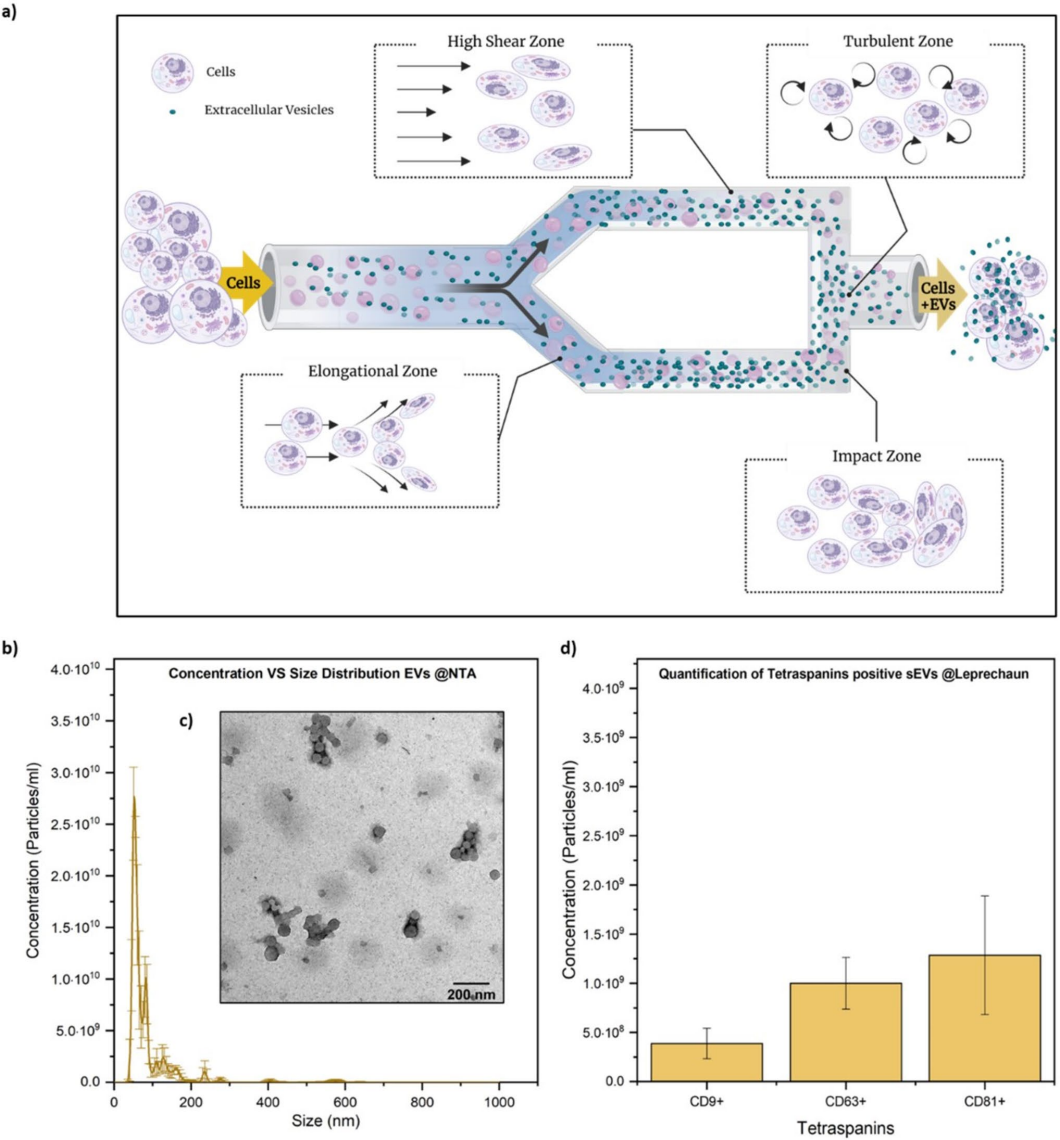
BV2 microglia-derived EV isolation and characterisation**. (a**) Working principle of the microfluidization approach to stimulate BV2 cells for EVs secretion. (**b**) Concentration versus size distribution of HPH-BV2-EVs and (**c**) their morphological appearance observed at the TEM microscope. Scale bar = 200 nm. (**d**) Biological quantitative assessment of Tetraspanins as general EV markers on BV2-derived-EVs

**Fig. 7:**
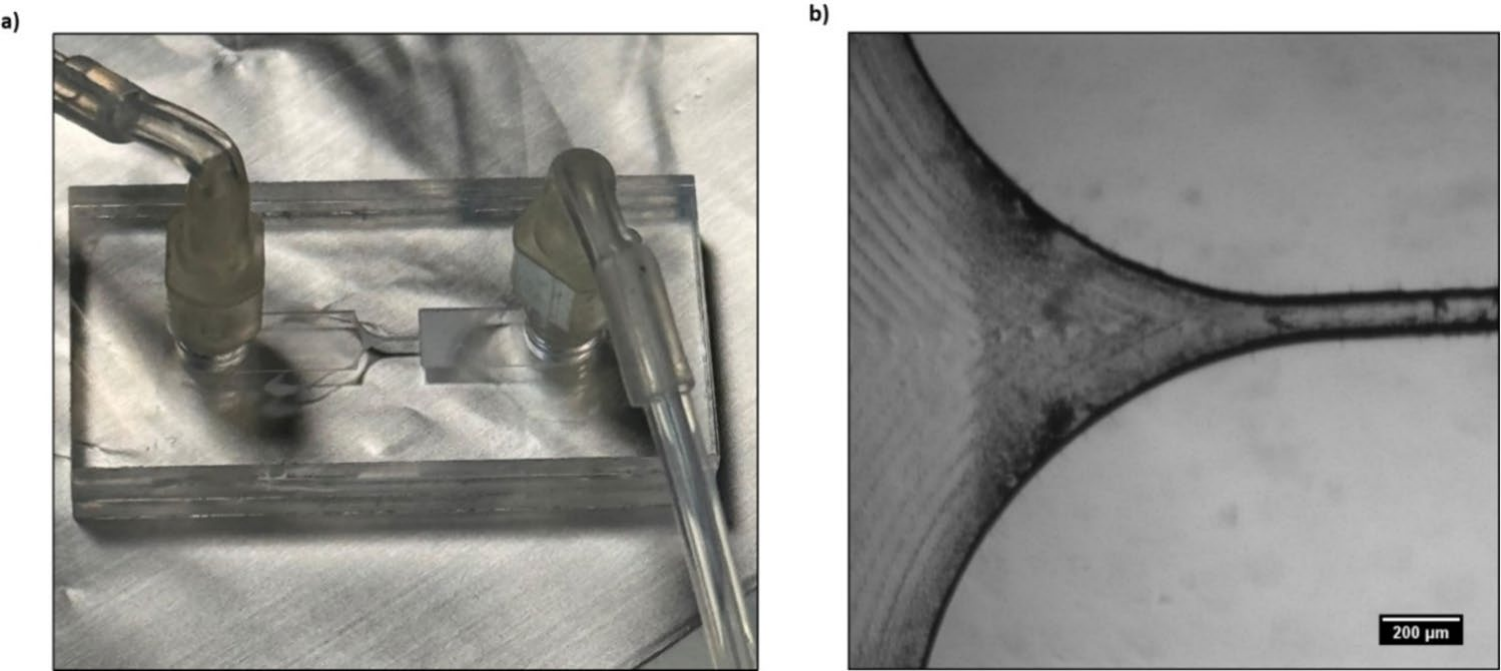
Hyperbolic elongational microfluidic device. (**a**) The set-up used to encapsulate synthetic miRNAs into BV2-HPH-derived-EVs is shown. (**b**) A zoomed-in view of the deformative compressive junction. Scale bar, 200 μm

The experiment described above, which employed synthetic miRNA mimics and transfection agent, demonstrated that specific AD EV-associated miRNAs are capable of activating hTLR8 signalling. To validate whether these miRNAs retain their functionality in a more biologically relevant context, we next engineered EVs to carry and deliver the miRNAs. This EV-based system more closely mimics natural intercellular communication and allows us to assess microglial activation in a setting that better reflects in vivo mechanisms of miRNA transfer and recognition. To this end, we made use of immortalized murine microglial BV2 cells as a source of EVs, due to their ability to closely mimic the functional and phenotypic properties of primary microglia. A high-throughput secretion of EVs was here performed through a novel method based on the microfluidization of cells. Due to the intrinsic fluidity of phospholipid membranes, which governs both the assembly of molecules into specific architectures and their curvatures, these structures exhibit a dynamic nature that is highly dependent on external conditions. Particularly under the influence of external forces, membranes deform by exhibiting a solid–fluid dualism, where their specific behavior is determined by the type of force applied and its direction. Therefore, the microfluidization approach grounded in the combination of shear-, elongational, collision-, and turbulent hydrodynamic forces established in a Y-shaped microfluidic valve of a High-Pressure Homogenizer (HPH), to stimulate a high-density pool of cells (Fig. 6a). As a result, a faster and high-throughput secretion of vesicles in flow was achieved compared to standard differential ultracentrifugation (see Supporting material figure S2), while also preserving cellular viability. Results from the nanoparticle tracking analysis (NTA) analysis (Fig. 6b) revealed a concentrated population of vesicles obtained with our microfluidic approach, with a total average concentration of 7.62 × 10^11^ particles/mL. These vesicles exhibited characteristic small dimensions, with mean and mode diameters of 85.9 and 54.0 nm respectively, a zeta potential of −32.5 mV, and common EVs morphological appearance as confirmed by TEM imaging (Fig. 6c). The resulting vesicles contained tetraspanins CD81 and CD63, while also exhibiting a high presence of CD9, indicating a significant biological content (Fig. 6d). Moreover, the vesicles were also evaluated for cytotoxicity, through a high-throughput monitoring of BV2 cell proliferation rates over 70 h upon contact with BV2-HPH-derived EVs using the xCELLigence system (see Supporting material figure S2). To engineer EVs and load them with synthetic miR-154-5p, miR-3150b-3p, miR-548b-3p or *let-7e,* we designed and fabricated a hyperbolic extensional microfluidic device by CNC micromilling technique, to finely exploit compressive and elongational forces to encapsulate the selected miRNAs into BV2-derived-EVs (Fig. 7). Immediately after the loading of synthetic miRNAs into the BV2-derived EVs, we quantified the encapsulated miRNAs by Nanodrop. Empty vesicles served as blank control. The encapsulation efficiencies (EE) were calculated based on the initial synthetic miRNA stock concentrations, taking into account the dilution factor applied during processing, as described in the supporting material 2. As shown in Supplemental material table S2, each encapsulation process yielded high efficiency, with encapsulation efficiencies (EE) values ranging from 76.4% to 94.1%, thus confirming the potential of the engineered EVs as valuable tools for miRNA delivery.

### miRNA Delivery by Microglia-Derived EVs Activates Microglia

**Fig. 8:**
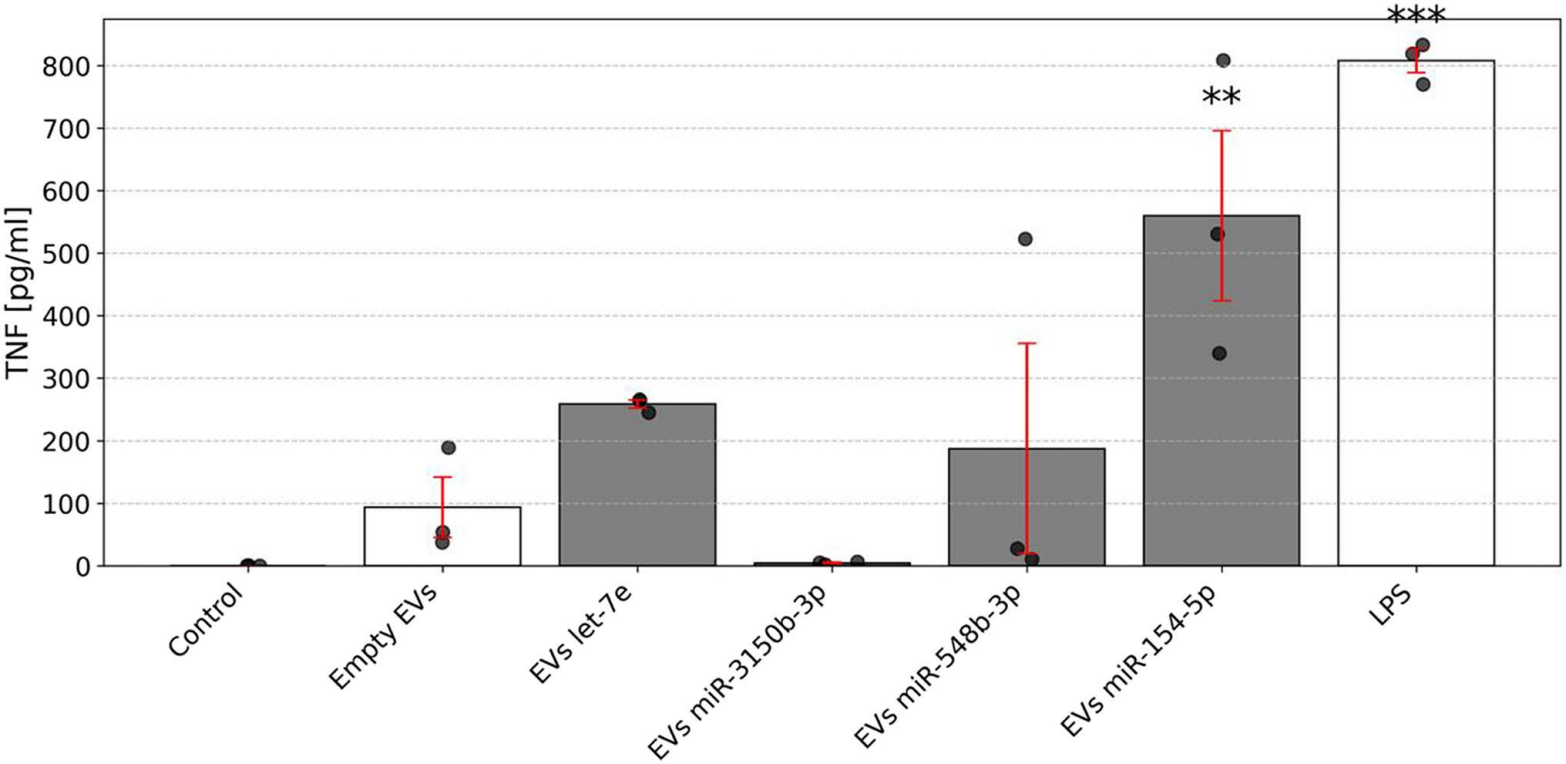
Selected EV-delivered miRNA induces TNF release from primary murine microglia**.** Primary microglia isolated from C57BL/6 mice were incubated with 7.5 × 10^9^ engineered EVs, as described above, containing individual miRNAs such as miR-3150b-3p, miR-548b-3p, miR-154-5p, or *let-7e*, for 24 h. Subsequently, TNF concentrations in the supernatant were quantified by ELISA. Unstimulated cells and cells treated with empty (miRNA-free) EVs served as negative controls, while LPS (100 ng/mL) was used as a positive control. Results are expressed as mean ± SEM. *n* = 3 independent experiments, where each experiment data point was a result of technical duplicate, averaged to yield a single value per experiment. Statistical analysis was performed using one-way ANOVA followed by Dunnett’s multiple comparisons test against the empty EV control group. *p < 0.05; **p < 0.01; ***p < 0.001

By engineering microglia-derived EVs to encapsulate specific synthetic miRNAs of interest, we aimed to evaluate whether these EV-delivered miRNAs are capable to induce activation of further microglia, thereby triggering an inflammatory response. This approach was considered to provide a more physiologically relevant context to assess the immunostimulatory capacity of microglial AD EV-associated miRNAs. To this end, we employed primary mouse microglia, which have been characterized as a valid model system for the investigation of innate immunity and inflammation in the CNS [6, 49]. Microglia were exposed to the EVs loaded with miRNAs associated with AD microglial EVs, namely miR-3150b-3p, miR-548b-3p, miR-154-5p, or *let-7e*. Empty EVs served as a negative control. After 24 h incubation, TNF concentrations in the supernatants of microglial cells exposed to the EVs were assessed (Fig. 8). EVs loaded with miR-154-5p significantly induced TNF release from microglia. In contrast, EVs containing miR-3150b-3p did not induce such a response. Supernatants from microglia exposed to EVs loaded with *let-7e* or miR-548b-3p showed an increasing trend of TNF release, however, those TNF release levels did not reach statistical significance. EVs without miRNA loading resulted in only slight TNF production (~ 100 pg/mL). LPS induced a significant TNF response, as expected. In summary, these data show that EVs containing selected microglia-derived AD-associated miRNA are capable of inducing an inflammatory response through microglial activation. Also, our data indicate that EVs can act as vehicles that shuttle distinct miRNAs into microglial cells.

## Discussion

**Fig. 9:**
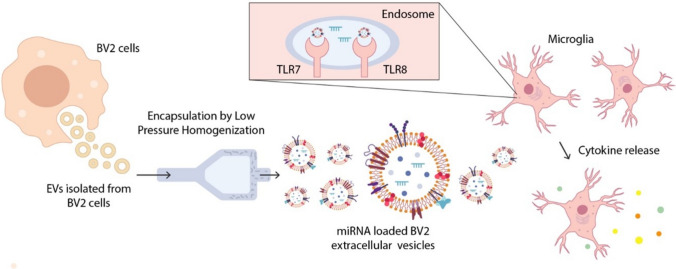
miRNA loading into EVs and its functional analysis in the context of neuroinflammation. This figure illustrates the workflow used to investigate the immunomodulatory function of AD-associated miRNAs delivered via EVs into microglial cells. EVs were first isolated from murine BV2 microglial cells and subsequently loaded with AD-associated miRNAs using a low-pressure homogenization technique to ensure efficient encapsulation. The resulting miRNA-loaded EVs were then applied to primary murine microglia. We hypothesize that internalization of EVs leads to the delivery of miRNA cargo into the endosomal compartment, where they can potentially interact with the RNA-sensing receptors TLR7 and TLR8. Potential activation of these receptors by specific miRNAs triggers microglial activation and the release of pro-inflammatory cytokines, such as TNF

Here, we show that EVs derived from microglia contain miRNAs that are capable of inducing an innate immune response in the context of AD pathology. By isolating putatively microglia-derived EVs from human CSF samples using an antibody directed against TMEM119 and performing miRNA profiling of the EV’s cargo, we identified a set of miRNAs specifically enriched in EVs of AD patients compared to control subjects. These miRNAs were functionally active molecules, as they directly activated hTLR8 and induced an inflammatory response in microglia. Furthermore, here, we engineered and validated a system for encapsulating such miRNAs in synthetic form into EVs for targeted delivery into microglia (see Fig. 9 for overview).

MiR-3150b-3p, miR-548b-3p, miR-154-5p, and *let-7e* associated with microglial AD EVs were predicted to function as TLR7/8 ligands, with our data confirming their selective activation of TLR8 in human TLR reporter cell systems. Mechanistically, our HEK reporter assays define receptor target engagement under standardized delivery conditions and show that the candidate miRNAs preferentially activate hTLR8 rather than TLR7. Accordingly, we interpret the TLR8-selective signal as evidence of intrinsic receptor preference for these miRNA sequences in the reporter context. This finding is of particular importance, as it suggests a mechanism by which endogenous miRNAs, shuttled via EVs, could directly activate and/or modulate innate immune responses and thereby contribute to chronic neuroinflammation, which is a hallmark of AD pathogenesis. Importantly, functional transfer of miRNAs into recipient cells generally requires a carrier, because “naked” small RNAs are highly charged and hydrophilic and therefore do not readily cross plasma membranes by passive diffusion. EVs possibly carry miRNA to the RNA-sensing TLRs located within the endosomal compartment of immune cells, such as microglia, and potentially further CNS cells, including astrocytes, and neurons, which also express these receptors [6, 38]. While the field has made strides in recognizing the importance of EV-mediated communication, the specific mechanisms governing how EV cargo is released and becomes functionally active within recipient cells are complex and multifaceted. EVs’ entry by endocytosis and cargo release via membrane fusion suggests that EVs exploit mechanisms similarly to viruses [50]. Such viruses exploit a low pH-induced change to induce fusion with acidified endosomal compartments [51, 52] and particularly, ssRNA viruses are recognized by TLR7[53]. This resemblance in entry and release pathways underscores a potentially conserved strategy between EVs and viruses in modulating host immune responses. Interestingly, the microglia-derived EVs’ cargo differs depending on the priming stimulus [14, 54, 55], which lends credence to the idea that microglial EVs actively participate in various diseases’ pathology. Important to mention, we focused on miRNAs as the central EV cargo feature because the study was designed and powered around EV-associated miRNA profiling, given their relative stability in biofluids, interpretability, and frequent use in EV-based biomarker work. Nevertheless, our small RNA sequencing datasets also contained additional non-coding RNA species, including mitochondrial-derived transcripts and other small ncRNA biotypes. We did not pursue a comprehensive analysis of these RNA classes, because this would require dedicated annotation and orthogonal validation and would shift the manuscript beyond its primary objective, which was to define and functionally test candidate EV-associated miRNAs in the context of AD.

A translationally significant result of our study was the successful engineering of EVs for miRNA delivery. Using a novel microfluidic encapsulation system with a hyperbolic extensional flow design, we achieved high-efficiency miRNA loading (up to 94%) into BV2 microglia-derived EVs, without compromising vesicle integrity or miRNA functionality. This approach marks a significant advance over conventional EV loading methods, offering a scalable and reproducible strategy for generating therapeutic EVs. Notably, these miRNA-loaded EVs were capable of activating primary murine microglia, as evidenced by TNF release following exposure. The strongest microglial response was induced by EVs loaded with miR-154-5p, which aligns with our previous work showing miR-154-5p as a direct activator of m/hTLR7 and hTLR8 thereby contributing to neuroinflammation including neuronal injury [48]. However, in contrast to this previous study [48], our current work employed patient-derived samples as miRNA source. Also, while our previous studies[6, 48, 56] and studies from other laboratories investigating the role of miRNAs in disease relied on synthetic oligoribonucleotides and transfection agents, such as LyoVec, to assess miRNA function in functional assays, our present work employed EV-mediated miRNA delivery. Accordingly, we view the transfection-based reporter experiments (Fig. 5) as defining receptor target engagement under standardized delivery, whereas the EV-based experiments (Fig. 8) address functional activity in a vesicular, more physiologically aligned delivery context. This strengthens the physiological relevance of the identified miRNAs and their potential involvement in disease, such as AD. However, in our current study, compared to miR-154-5p-loaded EVs loaded with other miRNAs such as miR-548b-3p and *let-7e* showed less pronounced, statistically not significant, effects in terms of microglial TNF release, whereas miR-3150b-3p was not capable of microglial TNF production at all. Nevertheless, several lines of evidence link *let-7e* found in microglial EVs to neurodegenerative disease. First, in our previous study, CSF *let-7e* copy levels were significantly higher in AD patients compared to healthy age-matched controls [57]. Notably, this increase in miRNA copy numbers was specific to AD, as patients with FTD did not show such an increase. In the same study we tested the neurotoxic potential of CSF-derived *let-7* miRNA. *let-7e* and other *let-7* miRNA family members present in CSF of patients with AD induced neuronal damage in vitro [58]*.* This supports the idea that in AD, *extracellular let-7e may function as a signalling molecule*, potentially activating microglia. In addition, analysis of neuron-derived exosomes in plasma found ***let-7e upregulated in AD*** and capable of provoking inflammatory responses, as *let-7e* from AD neural exosomes triggered IL-6 release from microglia [59]. This finding directly links AD-associated *let-7e* to microglial cytokine production, reinforcing its role in AD’s inflammatory pathology. The variability in cytokine responses to selected miRNAs may mirror the specificity of the respective miRNA acting as a signalling molecule. Also, the varying potential of miR-154-5p, miR-548b-3p, miR-3150b-3p, and *let-7e* to drive microglial cytokine release may underscore the importance of their specific sequence and secondary structure in determining TLR binding and signal transduction efficiency. Accordingly, TLR7 and TLR8 recognize single-stranded RNAs with particular GU-rich motifs and secondary structures that foster optimal receptor engagement. MiR-154-5p, shown here to strongly trigger microglial TNF release, may contain sequence features or structures that more readily fit the receptor’s binding pocket. In contrast, miR-548b-3p and *let-7e* exhibit comparably less pronounced effects in terms of cytokine release, which may be due to less favourable sequence motifs or structural elements, while miR-3150b-3p failed to activate microglia. This selective pattern of miRNA potency supports the concept that not all TLR-sensed RNAs are equally potent and that subtle differences in the miRNA’s molecular composition can significantly alter innate immune responses. Moreover, receptor sensitivity to specific miRNA motifs can vary across systems and species, which may explain, at least in part, the observed TLR8-selective response in our human TLR8 reporter cell setting [6, 60]. By transporting these miRNA ligands directly to microglia, EVs may help promote an environment conducive to TLR-mediated responses. Such a mechanism may underscore how EVs can drive or exacerbate neuroinflammation, positioning them as mediators of immune signalling in the CNS.

Especially from a translational standpoint, encapsulating miRNAs within EVs and validating their function in the context of immune signalling is crucial for several reasons. Firstly, EVs play a pivotal role in cell-to-cell communication by delivering their miRNA together with other EVs associated cargo to recipient cells. By examining miRNAs in the EV context, researchers should validate not only the presence of certain miRNAs but also their functional outcomes when internalized by target cells. This is key for understanding how miRNA-induced effects, such as immune activation or gene silencing, manifest in a physiologically relevant context. Studying free miRNAs in isolation or using transfection agents only partially mimics this process and may overlook the unique entry, trafficking, and release mechanisms that EVs employ.

Engineering EVs to carry specific molecules such as miRNAs or other therapeutic agents has got significant attention in recent years. Loading molecules into EVs can be achieved through various methods, including pre-isolation and post-isolation loading. Pre-isolation loading includes genetic manipulation, for instance transfecting donor cells with plasmids encoding therapeutic miRNAs or proteins, leading to their incorporation into EVs during biogenesis or using inducible promoters to control the expression levels of cargo molecules in donor cells. Post-isolation loading methods such as electroporation [61], sonication, or chemical transfection [62] can be inefficient or may damage EVs. It is well known that the aforementioned EVs post-loading encapsulation techniques result in low encapsulation efficiencies (EE) and potential damages for both EVs and model drugs. Recently, the use of hydrodynamic forces has been shown to be capable of achieving high EE for various molecules and EVs types [63–65]. In this study, a milder approach was developed, considering the process requirements for manipulating miRNA molecules. Therefore, a dedicated microfluidic system was designed to handle small volumes and promote the encapsulation of the candidate miRNAs by exploiting confined environments and elongational forces, resulting in high EE, while preserving miRNAs’ purity and stability, which effects were validated through biological assays.

In many pathologies, including neurodegenerative disorders, tumor progression, and immune dysfunction, EVs are a critical conduit for paracrine or even systemic communication. Confirming that a miRNA can still exert a functional effect once packaged within EVs adds a crucial layer of evidence for its role. Clinically, miRNA-loaded EVs also open up the potential for developing EV-based therapeutic approaches that rely on the stability, specificity, and biological activity EVs confer on their cargo.

This study has several limitations that should be acknowledged. First, although TMEM119-based immunocapture effectively enriched microglia-derived EVs, it remains possible that a subpopulation of EVs from non-microglial sources may still be present. TMEM119 was selected because it is widely used as a marker to distinguish resident microglia from other CNS-associated macrophage populations, supporting its use for cell type targeted enrichment strategies. Importantly, we interpret the resulting EV fraction as TMEM119-enriched rather than as definitively and exclusively microglial in origin. While targeted immunocapture is a practical approach for enriching low-abundance, cell type linked EV subpopulations from complex biofluids, it cannot fully exclude the possibility that a minor fraction of non-microglial EVs or non-vesicular material is co-isolated, particularly given the heterogeneity of CSF particles and EV surface epitopes. This limitation is consistent with broader community guidance emphasizing transparent reporting of EV isolation and characterization constraints and caution in assigning biogenesis or cellular origin when definitive proof is not feasible. Relatedly, the EV-marker western blotting in Fig. 2 is intended as qualitative and confirmatory evidence that immunocaptured material contains established EV-associated proteins, rather than as a primary quantitative readout. Immunocapture-based workflows often yield low material, and downstream western blot performance can be influenced by bead saturation, antibody affinity, epitope accessibility, and co-enrichment of matrix proteins. In our dataset, we did not have a robust normalization parameter across lanes (e.g., matched particle number, total EV protein input, or an orthogonal loading control), making quantitative comparisons of band intensity potentially misleading. In line with MISEV recommendations, we therefore avoid over-interpreting single-marker intensity differences as reflecting “more EVs” or “greater abundance” across capture conditions or diagnostic groups without rigorous normalization and yield assessment.

The apparent differences between CD63- vs TMEM119-captured fractions when probed for CD81 can be explained by EV heterogeneity and marker biology rather than by mutually exclusive capture. CD63 and CD81 are both commonly used tetraspanin-associated EV markers, but they are not uniformly expressed across all EV subtypes, and immuno-isolation using CD63 vs CD81 (or CD9) is known to enrich distinct small-EV subpopulations with different molecular profiles. Therefore, it is not necessarily expected that a CD63 pull-down will yield a uniformly “stronger” CD81 signal than a TMEM119-enriched pull-down; instead, the observed pattern may reflect (i) differential representation of CD81-high vs CD63-high EV subtypes in each immunocaptured pool and (ii) differences in capture efficiency and epitope accessibility across antibodies and bead-based formats. Furthermore, CD81 can appear as multiple bands with different apparent molecular weights, which may reflect different glycosylation states and/or more stable oligomeric/complexed forms that shift migration on SDS PAGE, and broader CD63 capture may co-enrich additional proteins that contribute to background or extra bands. Collectively, these considerations support our revised framing that TMEM119 immunocapture enriches an EV fraction consistent with microglial association, while banding patterns and relative intensities across markers should be interpreted as qualitative support for EV identity and heterogeneity rather than as direct quantification. Finally, although one could conceptually envision sequential capture (e.g., CD63-positive EVs followed by TMEM119 sub-isolation) to define a nested subpopulation, we did not pursue this approach because additional binding and/or elution and handling steps can reduce yield and potentially alter EV integrity or surface accessibility, an important practical constraint when working with limited-volume human CSF.

Second, the limited number of CSF samples may constrain the significance of small RNAseq results. Still, the functional relevance of the identified EV-associated miRNAs was confirmed through functional in vitro assays, lending robustness to the conclusions. Third, while TLR and microglial activation was robustly demonstrated in HEK TLR reporter cells and microglial cultures in vitro*,* respectively, the in vivo relevance of these findings, including the extent to which EV-associated miRNAs engage TLRs under pathophysiological conditions, remains to be investigated and validated in future studies. Fourth, the differential response of miRNAs in microglial activation assays, such as the lack of TNF release from microglia exposed to miR-3150b-3p despite hTLR8 activation in HEK TLR reporter cells, highlights the complexity of cell-specific signalling mechanisms. For instance, mouse TLR8 (mTLR8) was long considered non-functional due to its poor response to typical TLR8 agonists. This view has been revised by recent evidence showing that mTLR8 is indeed active and can influence neuronal growth and survival, for example by limiting neurite outgrowth and inducing neuronal apoptosis upon activation [66]. Moreover, although we prioritized functionally testing AD-enriched miRNAs with the strongest rationale, we recognize that inclusion of EV miRNAs that are down-regulated or not altered in AD would further strengthen conclusions about specificity. Future work will therefore incorporate such comparator miRNAs alongside sequence-scrambled controls to more stringently benchmark receptor engagement and inflammatory outputs across EV-delivered miRNA cargos. Finally, although our engineered EV platform demonstrated effective miRNA delivery, further research is needed to evaluate the impact of different EV doses, which may influence the magnitude and nature of the resulting immune responses. Addressing all of these aspects and further points will be critical for optimizing the therapeutic potential of EV-based RNA delivery systems.

## Conclusion

Our study demonstrates that putatively microglia-derived EVs from AD patients contain specific miRNA cargo, including miR-3150b-3p, miR-548b-3p, miR-154-5p and *let-7e.* Some of these miRNAs were found to act as ligands for hTLR8 and induce an inflammatory response from microglia. Microglia-derived EVs loaded with such miRNAs were functional by inducing TNF release from microglia. The identification of microglial EV-associated miRNAs and their potential role in neuroinflammation may help to open up a multitude of clinical applications, ranging from diagnostics and prognostics to targeted therapies and drug delivery systems. Also, our findings support a mechanistic role for EV-associated miRNAs in modulating the microglia-driven inflammatory response and may deepen our understanding how endogenous RNA signals may perpetuate neuroinflammation in AD. More broadly, our data point to the feasibility of using engineered EVs for miRNA delivery to immune cells within the CNS. Continued interdisciplinary research is required to enhance the translation of our findings into effective clinical tools, potentially improving the management and treatment of neuroinflammatory and neurodegenerative diseases.

## Supplementary Information

Below is the link to the electronic supplementary material.

Supplementary file1 (DOCX 152 kb)

Supplementary file2 (DOCX 380 kb)

Supplementary file3 (JPG 94 kb)

## Data Availability

Data and materials can be shared upon request.
