# Supplementary figures and images for: Microglia-Derived Extracellular Vesicles from Alzheimer’s Disease Patients Carry miRNAs Driving a Neuroinflammatory Response
Source: Mol Neurobiol. 2026 Feb 12;63(1):435. doi: 10.1007/s12035-026-05719-w (PMC12901236; doi:10.1007/s12035-026-05719-w)

*Supporting materials 1*

**HMC3 EVs cultivation and concentration**


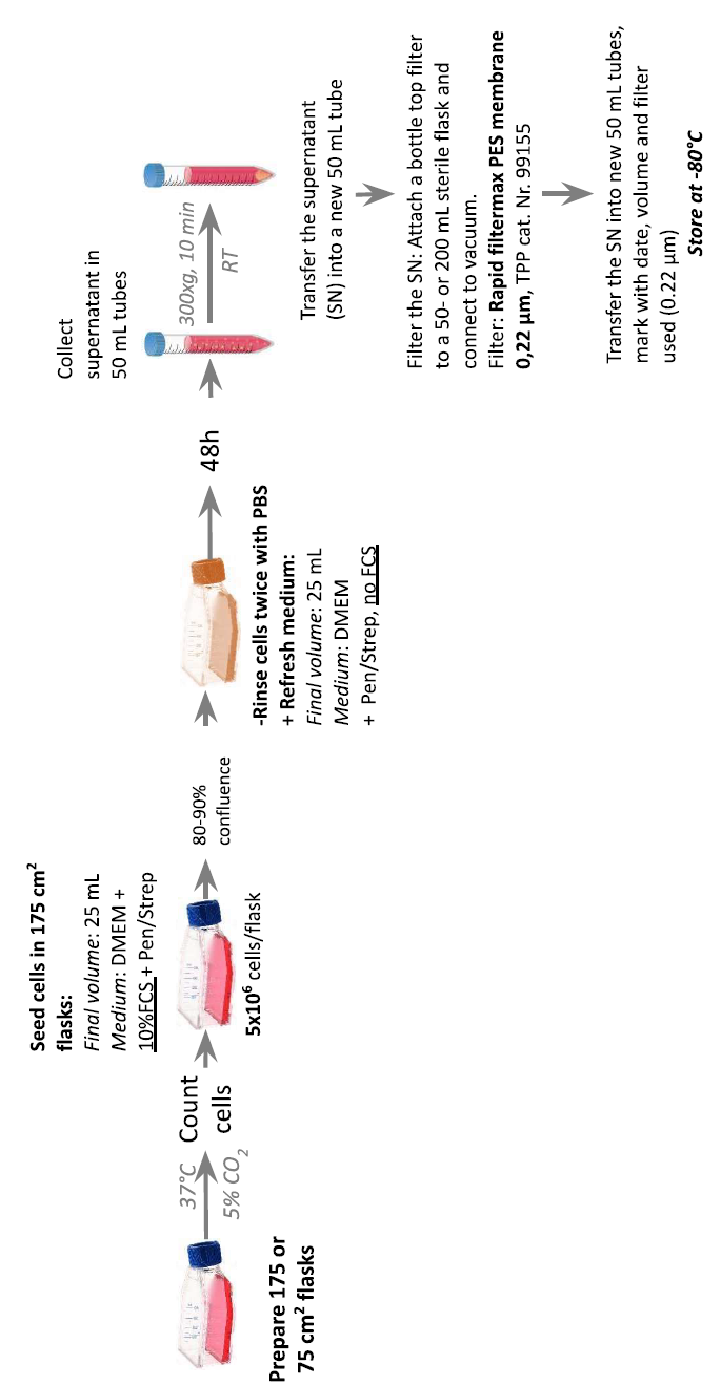

Supplement: Supplementary file 1 — Supplementary file1 (DOCX 152 kb) [file 12035_2026_5719_MOESM1_ESM.jpeg]

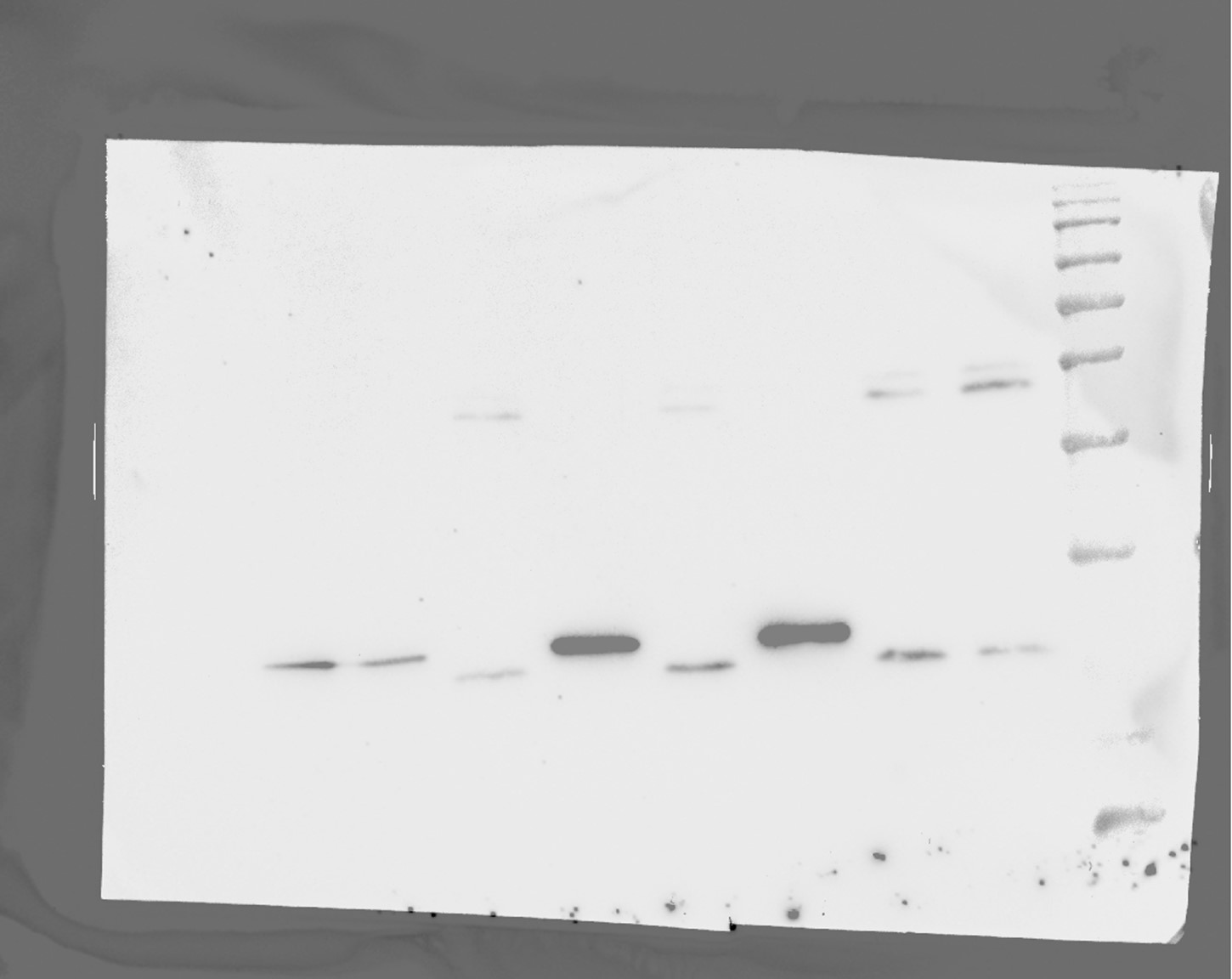

Supplement: Supplementary file 3 — Supplementary file3 (JPG 94 kb) [file 12035_2026_5719_MOESM3_ESM.docx]
