# Supplementary material for: Microglia-Derived Extracellular Vesicles from Alzheimer’s Disease Patients Carry miRNAs Driving a Neuroinflammatory Response
Source: Mol Neurobiol. 2026 Feb 12;63(1):435. doi: 10.1007/s12035-026-05719-w (PMC12901236; doi:10.1007/s12035-026-05719-w)
Supplement: Supplementary file 2 — Supplementary file2 (DOCX 380 kb) [file 12035_2026_5719_MOESM2_ESM.docx]

*Supporting materials 2*

**Enhanced yield of sEVs production by Microfluidization approach**

In this paragraph we compared the characteristics of sEVs obtained through standard differential ultracentrifugation (STD_dUC) and our high-pressure homogenizer (HPH) microfluidization approach.


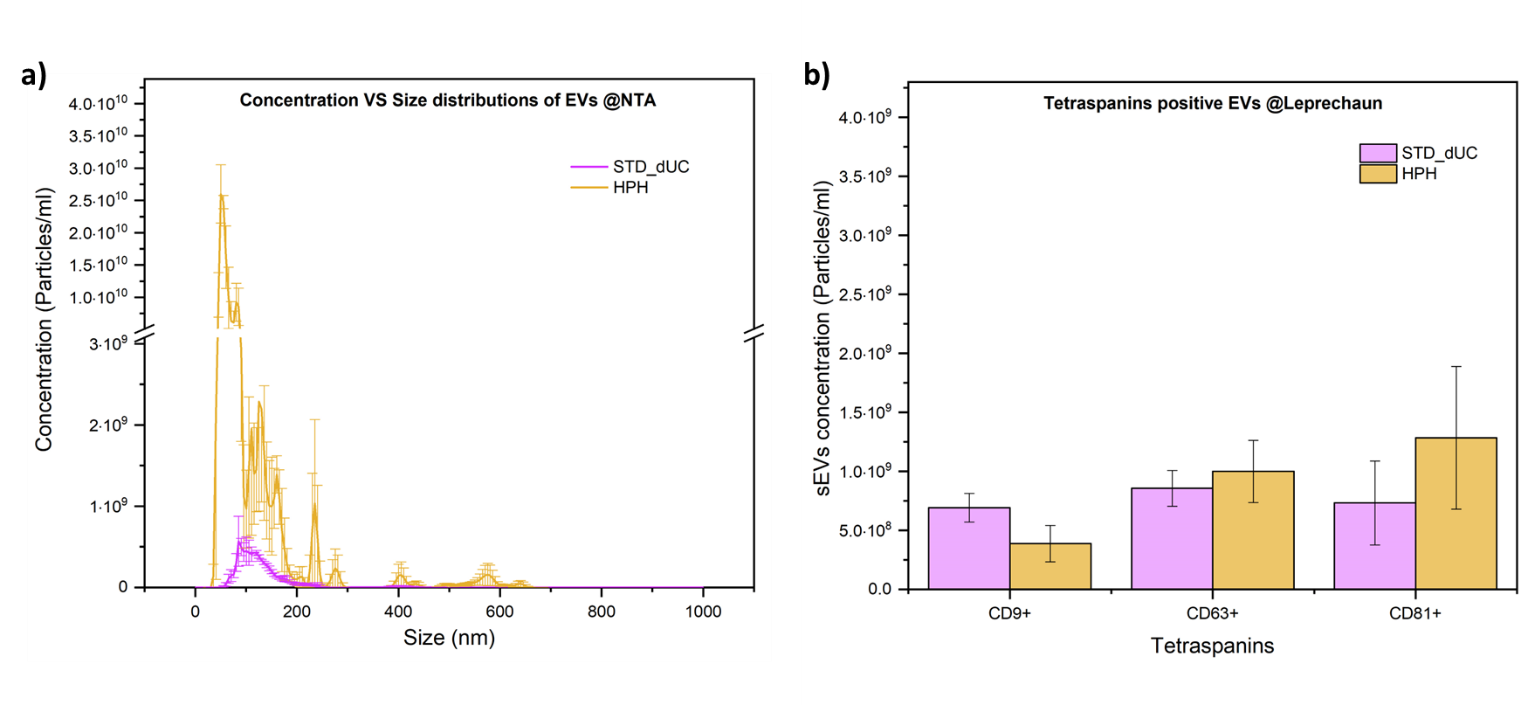


Figure 1: Comparison of the concentration VS Size distribution (fig. 1a) and the biological content of Tetraspanins content (fig. 1b) of BV2-derived-sEVs obtained with standard differential ultracentrifugation (STD_dUC) and High-Pressure Homogenizer (HPH) Microfluidization.

In fig.1a it is reported the concentration versus size distribution that was measured by nanoparticle tracking analysis (NTA). It highlighted that sEVs derived through HPH approach were an order of magnitude more concentrated with respect to sEVs obtained with STD_dUC, with an average concentration of 7.62x10^11^ and 3.78x10^10^ particles/ml, respectively.

Moreover, while HPH-derived sEVs displayed a mean and a mode sizes of 85.9 and 54.0 nm, STD_dUC-derived sEVs were characterized by an average size of 128.2 nm and a mode size of 95.8 nm, indicating that the Microfluidization approach stimulated the release of smaller vesicles with respect to the standard differential Ultracentrifugation method.

The biological content of vesicles in terms of tetraspanins (CD9^+^, CD63^+^, and CD81^+^) positive sEVs was then measured using the Leprechaun system, and a comparison of the results obtained with the two techniques are reported in fig.1b. As showed in the graph, the HPH-derived EVs exhibited a slightly higher concentration of CD63^+^ and CD81^+^. Despite not being significant in terms of increased biological content, it nonetheless confirms that the vesicles' nature was preserved during the HPH stimulation.

Overall, these findings demonstrated that the HPH approach allows for a higher yield of production of sEVs with preserved biological content, starting from a significantly smaller cell source, that was 7x10^6^ for HPH compared to 64x10^6^ cells for STD_dUC.

**High-throughput monitoring of BV2-HPH-derived sEVs cytotoxicity in vitro**


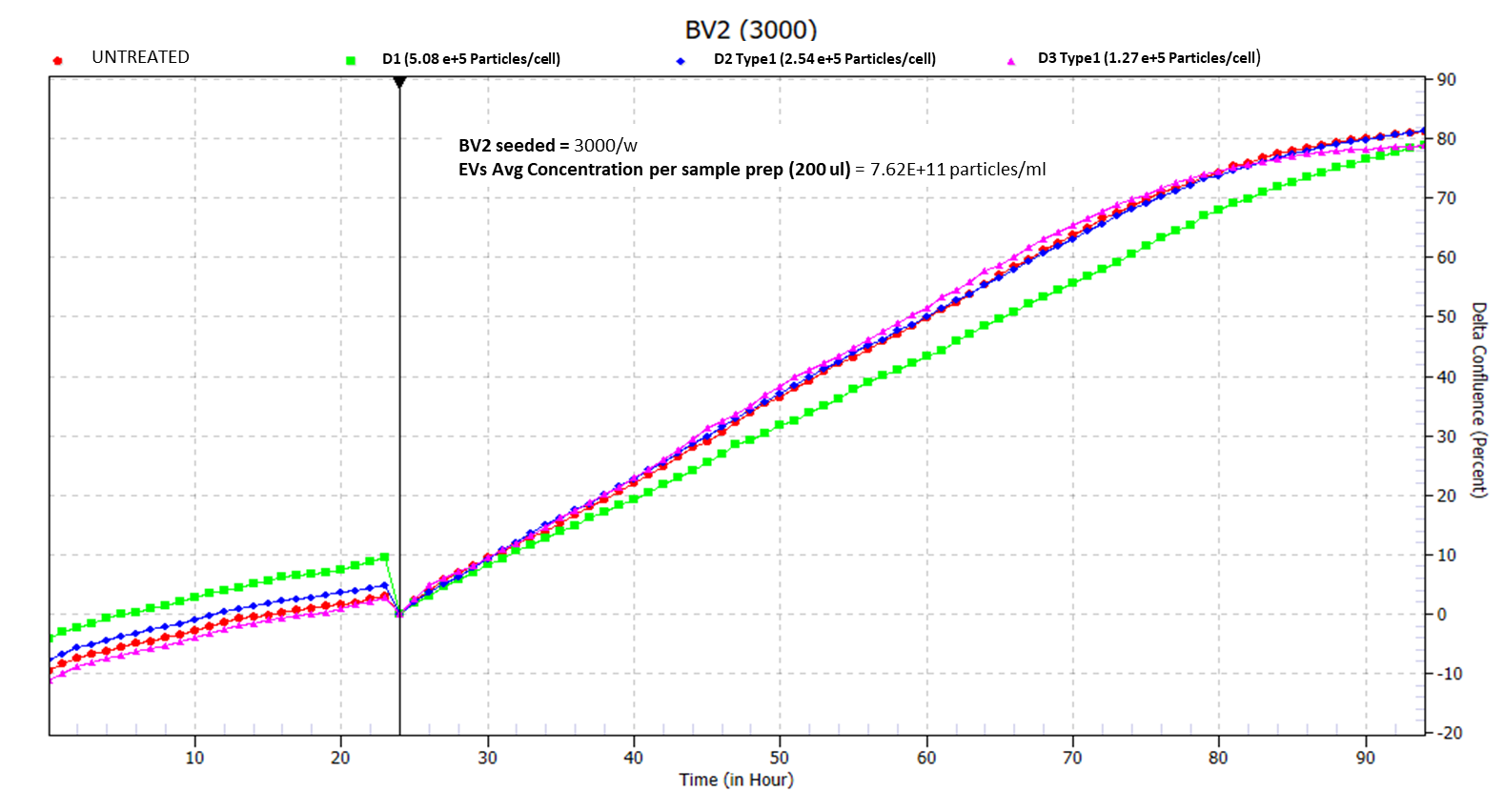


Figure 2: Cytotoxicity investigation of HPH-BV2-EVs measured through the real-time high-throughput monitoring xCELLigence RTCA system up to 94h. Three doses of HPH-BV2-EVs were put in contact with cells after 24h of seeding and proliferation curves were normalized for the concentration values at the contact time, so that Delta confluence (%) curves were displayed

In vitro cytotoxicity of BV2-HPH-derived sEVs was performed with a 70h real-time live imaging observation of BV2 cells in contact with vesicles by xCELLigence instrumentation, that was set in a brightfield-mode.

The data acquired with the xCELLigence instrumentation were processed with the segmentation adjustement that regulates the background-to-cells ratio equal to 1, while the adjust size (pixels) parameter was set at 0. Moreover, a filter on the minimum area recognized was set at 400 μm^2^, so that it could be possible to remove the cellular debris from the acquired objects.

In details, 3×10^3^ cells/well were plated in a flat and transparent 96-well plate (Corning, Costar, Merck) and incubated for 24h for adhesion. The day after, the well’s medium was refreshed and substituted with a suspension containing three doses of vesicles, namely D_1_=5.08x10^5^ particles/cell, D_2_=2.54x10^5^ particles/cell, D_3_=1.27x10^5^ particles/cell.

Following the incubation up to 70h, Cell viability and proliferation rates were assessed in terms of Delta Confluency (%) obtained by normalizing the curves to their confluence value at 24h, i.e. the time-point of the vesicles addition.

Results reported in fig.2 showed that doses D_2_ and D_3_ of sEVs had no impact on BV2 cell growth, as their proliferation curves overlapped the control. However, the highest dose D_1_ resulted in a slight but not significant inhibitory effect, as demonstrated by the small reduction of the confluence values over time. Overall, this analysis confirmed that the vesicles produced with our approach were safe, with all selected doses suitable for potential downstream applications.

**Encapsulation Efficiency (EE %) of engineered sEVs by microfluidics**

The total input volume that was loaded in the syringe before each microfluidic processing was equal to 1.2 ml, containing 75 µl of miRNA suspension. Therefore, we derived that the miRNA concentration of each candidate was diluted 1:16 from the initial stock concentration (C_i_) during the processing and we used these scaled values as theoretical concentrations expected for an encapsulation efficiency (EE)=100%. The encapsulation efficiency was then measured as

$$\mathrm{EE}\left( \% \right)=\frac{C_{f}}{C_{t}}\cdot100$$

With C_f_ being the miRNA concentration measured at Nanodrop after the encapsulation and C_t_ the theoretical value computed as stock concentration diluted 1:16.

|  | **Pre-Processing** | | | **Post-Processing** | | |  | |
| --- | --- | --- | --- | --- | --- | --- | --- | --- |
| **Sample (Blank=EVs)** | **C_i_** | **260/230** | **260/280** | **C_f_** | **260/230** | **260/280** | **C_t_** | **EE (%)** |
| EVs + miR-3150b-3p (Batch 1) | 1010 | 2.85 | 1.81 | 48.2 | 14.7 | 2.01 | 63.1 | 76.4 |
| EVs + let-7e-5p (Batch 2) | 1040 | 3.37 | 2.17 | 60.8 | 8.94 | 2.21 | 65.0 | 93.5 |
| EVs + miR-154-5p (Batch 3) | 1010 | 2.58 | 1.65 | 59.4 | 2.52 | 1.93 | 63.1 | 94.1 |
| EVs + miR-548b-3p (Batch 4) | 936 | 2.67 | 1.8 | 52.4 | 4.31 | 2 | 58.5 | 89.6 |

Table S1: Encapsulation Efficiencies measured at Nanodrop of miRNA candidates in HPH-BV2-EVs that were processed through the Hyperbolic extensional microfluidic device.
